# Self-Powered IIoT Steam-Trap Condition Monitoring Using Thermoelectric Energy Harvesting, Multimodal Sensing, and Machine Learning

**DOI:** 10.3390/s26216763

**Published:** 2026-10-26

**Authors:** Roger Malet, Lingyan Kong, Raúl Aragonés

**Affiliations:** 1 Department of Microelectronics and Electronic Systems, Universitat Autònoma de Barcelona, Bellaterra, 08193 Barcelona, Spain; roger.malet@uab.cat; 2 Alternative Energy Innovations S.L. (AEInnova), 08224 Terrassa, Spain; lingyan.kong@autonoma.cat

**Keywords:** steam trap, industrial internet of things, thermoelectric energy harvesting, ultrasonic sensing, LoRaWAN, machine learning, predictive maintenance, condition monitoring, steam leakage

## Abstract

**Highlights:**

**What are the main findings?**

- Histogram-based gradient boosting achieves 0.9853 test accuracy and 0.8822 macro-F1 against six rule-informed categories; stricter feature exclusion reduces macro-F1 to 0.4999.
- The runtime prototype applies registry-based eligibility rules to separate formal predictions, legacy shadow inference, and out-of-scope records.

**What are the implications of the main findings?**

- The results demonstrate agreement with engineering labels; independent fault labels and cross-device testing are required to establish diagnostic validity.
- Registry-based routing makes prediction eligibility explicit, but does not establish distribution-shift detection or generalisation to unseen devices.

**Abstract:**

Industrial steam-trap monitoring requires informative sensor measurements and reliable interpretation under practical power and communication constraints. This study examines a window-level machine learning workflow built on a previously reported thermoelectrically powered Industrial Internet of Things (IIoT) platform combining temperature and ultrasonic sensing with LoRaWAN communication. The dataset comprises 6354 windows from 2 monitoring-device series at 1 pharmaceutical facility, with 117 candidate engineered features. Six sensor-rule-informed pseudo-label categories are considered: normal, failed-open, failed-closed, degraded, excessive cycling, and short cycling. Random forest, extra trees, and histogram-based gradient boosting are compared using feature-exclusion configurations and a reported time-block evaluation. Histogram-based gradient boosting achieves reported test accuracy of 0.9853 and macro-F1 of 0.8822 with 108 retained features, and test macro-F1 decreases to 0.4999 under stricter exclusion retaining 81 features. These scores measure agreement with engineering labels rather than independently verified physical fault conditions. Normal and failed-closed are reported absent from the validation and test blocks, preventing complete six-class evaluation, and the recovered partitioning procedure does not establish global chronological or device-disjoint separation. A runtime prototype demonstrates registry-based eligibility control and visualisation of predictions and sensor context. The findings support a restricted pseudo-label classification workflow. Independent fault validation, cross-device evaluation, and measurement of the complete sensing and communication energy budget remain necessary to establish diagnostic validity and sustained energy-autonomous operation.

## 1. Introduction

Steam traps discharge condensate and non-condensable gases while limiting live-steam loss. Monitoring their condition is important because leakage and insufficient condensate discharge have different operational consequences and require different maintenance responses. Recent research has, therefore, investigated continuous, sensor-based monitoring as a complement to periodic inspection. Kim et al. combined statistical time-series features with a Transformer encoder–decoder model for steam-trap diagnosis in aluminium-processing and food-manufacturing facilities [1]. Their study illustrates the importance of linking sensor measurements to an explicitly documented diagnostic reference.

The interpretation of steam-trap measurements presents several challenges. Temperature describes thermal conditions, whereas acoustic measurements provide information about flow-related activity. Temporal descriptors can represent repeated discharge or changes in operating behaviour. These measurements must nevertheless be interpreted in relation to trap technology, installation, and operating conditions. Combining several feature families provides a rationale for richer observation but does not by itself demonstrate improved classification or independently validated fault diagnosis.

Recent diagnostic research also highlights the importance of label provenance. Kim et al. described labelling undertaken with steam-trap diagnostic engineers and supported by disassembly of selected suspected faulty traps [1]. This differs fundamentally from evaluating a classifier against labels generated from its own sensor-related inputs. Comparisons between studies must, therefore, consider the construction of reference labels, device populations, and evaluation design, rather than ranking reported accuracy alone.

Acoustic signal processing addresses a different part of the monitoring problem. Li et al. proposed steam-trap acoustic-emission denoising using sparrow-search-optimised variational mode decomposition and an improved wavelet threshold function [2]. Their evaluation concerns signal reconstruction and noise reduction rather than the six-category classification task considered here. This distinction matters because informative acoustic descriptors do not establish robustness to industrial interference. The present study uses precomputed acoustic features and does not independently evaluate their sensitivity to background noise or installation changes.

The broader predictive-maintenance literature also distinguishes data acquisition, preprocessing, feature selection, and decision modelling [3]. Within this framework, the present work addresses window-level condition classification and visualisation. It does not predict remaining useful life, estimate time to failure, or demonstrate maintenance scheduling benefits. Its comparison of tree-based models, therefore, concerns agreement with the available engineering labels, rather than validated prognostic performance or operational maintenance outcomes.

A separate requirement is supplying energy to distributed sensing nodes. Jiang et al. investigated thermoelectric self-powered wireless sensing using industrial waste heat [4], demonstrating the relevance of thermal harvesting to industrial sensor applications. However, energy autonomy depends on conversion, storage, and consumption throughout the operating cycle. Giuliano et al. characterised sensing and transmission consumption and investigated adaptive transmission intervals for battery-free LoRaWAN networks [5]. Although their study focused on photovoltaic harvesting, it provides a relevant example of evaluating the source, storage, and communication workload together. Harvester characterisation alone cannot establish the energy balance of a different sensing workload.

These studies provide complementary reference points rather than directly comparable performance benchmarks. Steam-trap diagnosis with field-supported labels [1], acoustic denoising [2], thermoelectric sensor powering [4], and adaptive battery-free communication [5] address different targets and use different validation evidence. The present workflow combines descriptors from an existing sensing platform with pseudo-label classification and registry-based runtime control. Its principal evidential limitation is that neither high label agreement nor successful routing establishes independent fault recognition, noise robustness, or workload energy autonomy.

Our previous publications reported the heat-powered IIoT architecture [6] and its application to temperature-and-ultrasound steam-leak monitoring [7]. The sensing hardware, thermoelectric platform, and laboratory characterisation summarised in Section 3 originate from those studies and are included as background—they are not new hardware experiments in the present work. The contribution presented here is the analysis of precomputed window-level data using six engineering pseudo-label categories, comparison of three tree-based classifiers, assessment of two feature-exclusion configurations, and demonstration of registry-based routing and a dashboard prototype. These analytical results extend the earlier sensing work, but do not constitute independent validation of six physical fault states or a new verification of the complete node energy budget.

Related work also extended thermoelectric powering to Narrowband Internet of Things (NB-IoT) monitoring in the oil and gas industry [8]. That hardware and communication study provides additional platform context, rather than validation of the six-category classifier evaluated here. Specifically, this study compares three tree-based classifiers using precomputed thermal, acoustic, pulse/cycling, and statistical descriptors. The targets comprise six project-specific engineering categories: normal, failed-open, failed-closed, degraded, excessive cycling, and short cycling. They are rule-informed pseudo-labels rather than independently confirmed physical fault records. Consequently, the evaluation measures agreement with this operational taxonomy. It does not establish that the classifier diagnoses six physical fault states independently of the labelling procedure.

The studies discussed above address different questions: sensor-based steam-trap diagnosis with field-supported labels [1], thermoelectric powering of wireless sensors [4], and energy-aware communication in battery-free networks [5]. Our earlier publications established the sensing and power-conversion platform [6,7], but did not evaluate the six-category window-level classification workflow examined here.

The study addresses three questions: (i) how the three candidate classifiers compare in agreement with the available engineering labels, (ii) how classification performance changes under additional feature exclusion, and (iii) how the runtime implementation applies a predefined device-eligibility policy. The corresponding contributions are as follows:

- A comparison of random forest, extra trees, and histogram-based gradient boosting on the available window-level dataset, evaluated using accuracy and macro-F1 against six rule-informed engineering categories. This assesses label agreement within the reported partition, rather than independently verified fault-diagnosis accuracy.
- A comparison of the 108-feature and 81-feature configurations that quantifies sensitivity to additional feature exclusion. This assesses dependence on the retained representation but does not identify individual feature contributions or establish independence from the labelling rules.
- A demonstration of registry-based runtime routing that reports the numbers of records assigned to formal prediction, legacy shadow inference, and rejection. This verifies application of the predefined eligibility policy, rather than detection of distributional shifts or validity on unseen devices.

The dashboard provides an interface for inspecting these outputs and their sensor context. It is presented as an implementation component rather than a separately validated scientific advance.

The remainder of the paper describes the steam-trap condition framework, the previously reported sensing platform, the available feature representation, the classification methodology, the results and their limitations, and the validation required before broader diagnostic deployment.

## 2. Steam-Trap Operation, Failure Modes, and Monitoring Requirements

### 2.1. Role and Types of Steam Traps

Steam is used as a heat-transfer medium across process industries. Established studies and technical guidance describe energy use in steam generation, distribution, and condensate recovery [9,10]. The main elements of a steam circuit are as follows:

- Steam boiler. It is the element that converts clean water into steam thanks to several heating elements (electric resistors, fuel, gas, etc.).
- Steam pipelines. Their mission is the transportation of steam in all processes.
- Pumps. They are used for pumping water condensed to the boiler.
- Steam traps. In a steam pipeline, they are typically installed every 30 to 50 m depending on factors like pipe size and operating conditions.
- End of steam pipelines. The steam trap (Figure 1) allows to purge condensate before it re-enters the system.

Steam traps can broadly be grouped into mechanical, thermodynamic, and thermostatic devices. Mechanical traps respond primarily to the density difference between steam and condensate. Thermodynamic traps operate through flow and pressure effects and commonly exhibit repeated opening and closing behaviour. Thermostatic traps respond to temperature differences between steam, condensate, and non-condensable gases. Consequently, the same measured signal should not necessarily be interpreted identically for all trap types (Table 1).

Technical guidance describes steam traps as devices for removing condensate and non-condensable gases while retaining steam [11]. Commercial monitoring also combines thermal and ultrasonic observations: Armstrong describes this approach for its SteamEye system [12]. These manufacturer resources provide operating and product context, rather than independent validation of the present classifier. ISO 5117:2023 specifies production and performance characteristic tests for automatic steam traps [13]—the present study does not claim testing to that standard or derive its six-state taxonomy from it.

Technical guidance also identifies limitations of individual diagnostic methods. TLV describes visual, thermal, and ultrasonic inspection and explains that temperature alone cannot reliably distinguish flash steam from leaking live steam [14]. These limitations support interpreting thermal and acoustic evidence together, without establishing that a combined classifier is more accurate. Peer-reviewed work addresses complementary diagnostic requirements: Kim et al. describe engineer-supported fault labelling and disassembly of selected suspect traps [1], whereas Li et al. evaluate acoustic-emission denoising [2]. Neither result validates the numerical boundaries of the six categories used in this study.

### 2.2. Failure Modes

The six-state taxonomy was adopted specifically for the analytical framework developed in this project. It is not presented as a standardised taxonomy or a universally recognised industrial maintenance classification. Normal, failed-open, and failed-closed reflect conventional operating interpretations, while degraded, excessive cycling, and short cycling extend the framework to intermediate and dynamic sensor patterns. Their inclusion was intended to provide a more detailed operational description than binary leak detection. However, this engineering motivation does not establish that the six categories are physically distinct, mutually exclusive, or reproducibly assigned.

The available thesis describes their construction through combined interpretation of thermal, acoustic, and pulse/cycling information [15], but does not document a formal taxonomy development protocol, complete numerical assignment rules, or independent validation. Table 2, therefore, presents the physical rationale for the categories rather than verified diagnostic boundaries. The classifier evaluation measures agreement with this project-specific labelling framework—it does not validate the taxonomy itself.

For normal operation, measurable criteria would need to specify an expected range of thermal, acoustic, and cycling behaviour under documented operating conditions. Failed-open and failed-closed criteria would need to distinguish sustained steam passage from insufficient condensate discharge, including alternative explanations for similar sensor patterns. Degraded would require an explicit boundary separating intermediate abnormalities from normal operation and the other categories. Excessive cycling and short cycling would require defined cycle events, observation intervals, and reference limits for frequency and duration. The original numerical criteria and overlap-resolution rules were not recovered from the available materials. The present categories must, therefore, be interpreted as sensor rule pseudo-labels whose reproducible assignment and independent physical validity remain unresolved.

Excessive cycling and short cycling describe related temporal characteristics. Excessive cycling conceptually concerns cycle frequency, f = N/T, where N is the number of complete operating cycles observed during an interval of duration T. Short cycling concerns short cycle durations or inter-cycle intervals, Δt_i_ = t_i+1_ − t_i_, using a consistently defined cycle event. High frequency and short intervals may occur simultaneously, so distinguishing these categories requires trap-specific reference limits and an explicit rule for resolving overlap. These definitions provide physical context rather than a reconstruction of the dataset’s labelling algorithm: the available documentation does not establish the mapping between recorded pulses and complete cycles, the numerical class boundaries, or their precedence. The two outputs should, therefore, be interpreted as engineering pseudo-label categories whose physical separation remains unvalidated.

These states should be interpreted as condition-monitoring indicators rather than direct maintenance work orders. In particular, degraded and cycling-related states may overlap with normal behaviour depending on trap technology and operating conditions. Final maintenance decisions should incorporate plant context, device history, and, where possible, field confirmation.

### 2.3. Environmental and Economic Motivation

Steam loss and insufficient condensate discharge motivate condition monitoring because they can affect energy use and process operation. Recent steam-trap diagnostic research has investigated these issues in industrial settings [1]. However, the present study does not measure leakage rates, maintenance outcomes, or safety incidents. Classification against engineering pseudo-labels, therefore, cannot establish improved efficiency, safer operation, or avoided emissions.

Thermoelectric harvesting provides a technical rationale for reducing dependence on primary batteries where sufficient thermal energy is available [4,6,7]. Its operational benefit depends on the complete energy budget, installation conditions, and reliability of the sensing system. Battery replacement effort, total maintenance requirements, and lifecycle environmental impacts were not quantified in this study.

The research gap is, therefore, the integration of (i) process-heat-powered sensing, (ii) multimodal thermal and acoustic observation, (iii) long-range IIoT communication, and (iv) scope-aware multi-state machine learning in one monitoring workflow. This combination is intended for distributed industrial assets where battery replacement and wired instrumentation can become significant lifecycle constraints.

The present work addresses a complementary problem: how to obtain continuous multimodal data autonomously while extending the analytical output from leakage detection to a defined six-state condition-monitoring framework. Unlike approaches that assume externally powered sensor infrastructure, the proposed workflow couples process-heat-powered sensing with long-range IIoT communication and a scope-aware machine learning layer.

### 2.4. Physical Signatures of Steam-Trap States

The physical observability of a steam-trap condition depends on the interaction between temperature, flow, pressure, and valve dynamics. Under normal operation, the trap removes condensate while limiting live-steam passage. The upstream and downstream thermal conditions, therefore, differ according to the trap design and process operating point. When a trap remains open, the downstream side can approach the upstream thermal condition because steam passes continuously through the device. Conversely, a blocked or closed trap can cause condensate accumulation and a reduction in the expected downstream thermal response.

Temperature alone is not always sufficient to distinguish these states. Hot condensate can undergo flashing after a pressure reduction, producing thermal conditions that resemble steam flow. The previously published steam-trap sensing study combined inlet/outlet PT100 measurements with ultrasonic sensing. It reported that ultrasonic activity around the 30–40 kHz region was useful for identifying steam leakage while avoiding part of the industrial noise observed at lower frequencies. The ultrasonic microphone used in the prototype had a 50 kHz bandwidth and the acquisition stage operated at 160 kHz sampling. This multimodal principle motivates the feature design in the present study. Rather than asking one sensor to identify a failure, the machine learning model receives complementary evidence: thermal level and difference, acoustic intensity and spectral behaviour, and temporal pulse or cycling activity. The expectation is not that every feature is useful for every class. Instead, the combination should provide a richer representation in which different physical signatures become available to the classifier.

These factors were not systematically incorporated as documented contextual inputs or evaluation strata in the present analysis. The available records do not support performance comparisons by trap technology, pressure, load, condensate condition, installation, or noise level. Registry-based device eligibility does not resolve this limitation: it identifies designated device series but does not assess their operating conditions. The reported results, therefore, apply to the available feature representation and engineering labels within the evaluated dataset, rather than demonstrating robustness across these sources of variability.

## 3. Materials and Methods: Autonomous Thermoelectric Iiot

This section summarises the previously reported thermoelectric sensing platform that provides the hardware context for the present analytical workflow [6,7]. It is not a complete hardware replication specification or a new experimental verification of energy-autonomous operation. The available materials do not provide a consolidated component inventory, complete acquisition and communication configuration, or measured whole-node energy budget for the workload considered here. Accordingly, the architecture description and earlier TEG characterisation should be distinguished from evidence that the complete sensing, local processing, and radio workload can operate sustainably under field conditions.

### 3.1. System Overview

As shown in Figure 2, the architecture comprises five functional layers. The first is the energy harvesting and sensing layer attached to the steam system. The second is the embedded electronics layer, which acquires and conditions sensor signals, performs local processing, and provides LoRaWAN communication. The third is the LoRaWAN gateway. The fourth is the external data-processing platform, where window-level records are prepared and analysed by the machine learning workflow. The fifth is the visualisation interface for inspecting prediction status and sensor context.

The node is intended to be installed externally, avoiding intrusive modifications to the steam path. The temperature interface provides thermal information while the ultrasonic channel provides information about high-frequency flow activity. The architecture can additionally use pulse or cycle indicators derived from the acoustic signal. This combination is important because no single physical variable fully describes all steam-trap states.

LoRaWAN is a low-power wide-area network (LPWAN) technology [16] and provides the communication layer of the previously reported sensing platform [6,7]. However, the available materials do not establish the transmission power, spreading factor, payload size, transmission schedule, packet-delivery rate, gateway distance, or energy per communication cycle for the records analysed here. The approximately five-minute transmission interval described in the earlier steam-trap implementation [7] is a historical platform parameter, not a verified acquisition or transmission cadence for the present dataset. The supplied Python 3.13.5 scripts process precomputed window records and do not configure or evaluate the radio link. Consequently, the present study does not quantify communication reliability or its contribution to the node energy budget.

### 3.2. Thermoelectric Energy Harvesting

The thermoelectric power converter and laboratory characterisation summarised in this subsection were previously reported in [7]. The measurements, test-bench description, and associated illustrations are included to explain the hardware foundation of the present analytical workflow—they were not generated as new experiments for this study.

All tests were conducted entirely in AEInnova’s laboratory, a spin-off of the Microelectronics Department at the Autonomous University of Barcelona (Figure 3), where the authors of this paper are principal founders. The laboratory characterisation summarised here belongs to the earlier hardware study, reported in 2024. It is separate from the field monitoring campaign conducted between August 2025 and March 2026, whose window-level records are analysed in the present manuscript.

For the complete characterisation, a test bench was developed, consisting of a 2-inch pipeline heated by a 150 W resistor and a support structure for the pipeline. The test bench includes a PID controller and a precise PT100 temperature sensor to ensure accurate temperature control of the pipeline surface. For the measurements, the temperature was increased in 10 °C increments, with a 20-min stabilisation period for the thermoelectric generator (TEG) surface temperature at each step.

The energy source is the temperature difference between the hot steam system and the surrounding environment. A thermoelectric generator converts part of this thermal gradient into electrical energy through the Seebeck effect. The usable electrical power depends on the thermoelectric material, hot- and cold-side temperatures, thermal interfaces, heat-sink design, and electrical operating point.

The thermoelectric sensing platform described in the earlier steam-trap study used a dedicated thermoelectric module coupled to a thermal interface and radiator. Characterisation showed that the open-circuit voltage increased with source temperature and temperature difference. In the reported experiments, the system was designed to supply the embedded electronics and sensors without a primary battery. The power management stage includes DC/DC conversion and energy storage, allowing intermittent sensing and radio transmission even when instantaneous harvested power is lower than the peak system demand. Power generation results are presented in Figure 4. Table 3 summarises thermoelectric performance [7].

A key design principle is, therefore, energy-aware duty cycling. Sensing, feature extraction, and communication are scheduled so that the average energy demand remains compatible with the available harvested energy. A supercapacitor can buffer the difference between slow thermal energy accumulation and short communication bursts.

#### 3.2.1. Thermoelectric Conversion Model

For a first-order representation, the open-circuit voltage generated by a thermoelectric generator can be expressed as:

V_OC_ ≈ S_eff_ × ΔT (1)

where S_eff_ is the effective Seebeck coefficient of the module and ΔT is the temperature difference between the hot and cold sides.

Under load, the delivered electrical power depends additionally on the internal electrical resistance and the operating point imposed by the power management circuit. The maximum-power condition is, therefore, not equivalent to the open-circuit voltage condition.

The previous steam-trap study characterised the TEG on a 2-inch heated pipe test bench using a 150 W resistor, a PID controller, and a PT100 reference. The hot-side temperature was increased in 10 °C increments with approximately 20 min stabilisation at each step. Measurements covered hot-side temperatures from 50 °C to 170 °C and ambient temperatures from 15 °C to 40 °C. The maximum reported open-circuit voltage was 3.687 V at 170 °C hot-side temperature and 15 °C ambient temperature. The maximum reported generated power was 1.034 W under the same condition [7].

The reported hot-side and ambient temperatures do not establish the actual temperature difference across the thermoelectric module, because the cold-side temperature depends on heat transfer through the radiator and installation. The maximum-power operating point, therefore, cannot determine the minimum thermal gradient required to start or sustain the sensing node. Cold start and sustained operation also represent different requirements: starting depends on the converter and initial storage state, whereas continued operation depends on harvested energy, workload, and usable stored energy.

The present study does not report cold-start time, minimum operating gradient, recovery after power interruption, or supercapacitor-supported operating duration. Operation during low-load and shutdown periods was not independently evaluated. Consequently, the earlier laboratory characterisation should not be interpreted as evidence of uninterrupted monitoring across these conditions.

The reported temperature increments and stabilisation periods describe the earlier test procedure, but they do not quantify measurement uncertainty or repeatability. Calibration records, instrument uncertainty specifications, the number of independent repetitions at each operating point, and the corresponding variability were not available in the materials recovered for this revision. Consequently, uncertainty intervals and error bars cannot be reconstructed reliably for the summarised thermoelectric results. The reported voltage and power values are retained as results attributed to [7], without implying that their measurement uncertainty was independently assessed in the present study.

These results are important for the present paper for a reason that goes beyond the peak power value. The ML system requires repeated acquisition, feature extraction, and wireless transmission. A process that can provide a sustained temperature gradient can, therefore, act as the energy budget for the complete sensing pipeline. In opposition, the relevant deployment quantity is the minimum sustainable harvested power under the actual thermal installation, not the maximum laboratory power. The distinction should be retained when moving from laboratory characterisation to field deployment.

#### 3.2.2. Energy Budget and Duty-Cycled Operation

The autonomous node should be analysed as an energy-balance problem. Let P_H be the average harvested electrical power, P_S the average sensing power, P_E the embedded-processing power, P_C the average communication power, and P_L the losses in power conversion and storage. A sustainable operating point requires:

P_H ≥ P_S + P_E + P_C + P_L.(2)

Because communication is burst-like, the supercapacitor or storage element decouples the instantaneous radio demand from the slower thermal harvesting process.

This architecture also changes the design logic of the ML system. A conventional powered sensor may acquire raw waveforms continuously and transmit large data volumes. A heat-powered node cannot assume the same energy budget. Feature engineering, therefore, becomes an architectural function: the node can reduce high-rate measurements to compact descriptors and communicate those descriptors rather than continuous raw signals. The resulting system simultaneously reduces radio energy and creates a structured dataset suitable for tabular machine learning.

The previous implementation transmitted data approximately every five minutes and used LoRaWAN as the long-range communication mechanism. The present paper does not claim that this interval is universally optimal; rather, it demonstrates the principle that the sensing workload and communication schedule must be designed together with the available thermal energy [7].

The energy-balance equation states a requirement for sustainable operation rather than a measured result. A quantitative assessment would require usable harvested energy at the regulated supply, sensing, and local-processing energy per acquisition cycle, LoRaWAN transmission and reception energy, standby consumption, and conversion and storage losses over a representative operating interval. Communication peaks and any retransmissions must also be accommodated by the energy buffer. These quantities were not measured together for the present workload. The earlier peak TEG power, therefore, cannot establish sustainable operation under field thermal conditions. The classifier and dashboard run in the external analytical layer and are distinct from local processing on the sensing node. Batteryless describes the powering architecture, and continuous energy-autonomous operation of the proposed workload remains unverified.

#### 3.2.3. Acoustic and Ultrasonic Observation

Ultrasonic sensing provides a complementary observation channel because high-frequency acoustic energy can be associated with turbulent or high-velocity flow through the trap. The prototype used a 50 kHz bandwidth ultrasonic microphone. Because the microphone membrane is sensitive to temperature, the mechanical implementation separated the microphone from the hot trap and used an aluminium waveguide to transfer acoustic energy while reducing the thermal exposure. This mechanical detail is relevant to autonomous sensing because sensor survivability is part of the overall system reliability. The previous experiments showed that the acoustic signal could reveal leakage episodes that were not sufficiently unambiguous from temperature alone. In particular, the manuscript reports a characteristic response around 37 kHz and, therefore, focuses on a 30–40 kHz band to reduce interference from lower-frequency industrial noise. In the present ML pipeline, the acoustic signal is not treated as a single threshold. Its RMS level, band ratio, peak value, peak frequency, and temporal statistics are transformed into features, allowing the model to learn combinations of acoustic and thermal evidence.

## 4. Materials and Methods: Data Acquisition and Feature Engineering

### 4.1. Device Scope and Dataset

The current-scope dataset comprises 6354 precomputed window-level records from two LS 2.0 monitoring-device series collected at one pharmaceutical facility in Barcelona between August 2025 and March 2026. The DatasetZ series contributes 4932 windows and the DatasetI series contributes 1422 windows. These counts describe monitoring-device series and analysis windows—they do not establish the number of distinct physical steam traps, independent fault events, or maintenance-confirmed conditions.

The available documentation does not establish a complete device-to-trap mapping, trap technologies, operating pressures and loads, sensor-specific acquisition frequencies, exact window duration, stride or overlap, or missing-data rates by signal and device. These parameters cannot be inferred from the window counts or the ultrasonic waveform sampling rate reported for the earlier hardware. Their absence limits reproduction of data acquisition and feature construction, as well as interpretation of the cycling descriptors.

The available dataset description lists 4449 training, 954 validation, and 951 test windows. These counts differ from those generated by the recovered time-block script and cannot be confirmed as the partition underlying the reported chronological metrics. Section 5.5 describes the available partitioning procedure and this unresolved provenance limitation.

### 4.2. Window-Level Representation

Continuous sensor streams are transformed into analysis windows. Each window is represented by a set of descriptive variables that capture thermal level and variation, acoustic activity and spectral characteristics, pulse and cycling behaviour, and statistical properties. This representation reduces the dimensionality of continuous sensor streams while preserving information relevant to the operating state.

#### 4.2.1. Temporal Window Rationale

A window-level sample is preferable to treating every raw measurement as an independent observation because steam-trap behaviour is dynamic. A single temperature can be compatible with several operating conditions, whereas a sequence can reveal a stable level, repeated pulses, a gradual thermal drift, or a transition between states. The dataset description [15] treats each analysis window as one machine learning sample and extracts temperature, acoustic, pulse, and statistical descriptors from that interval.

Windowing also reduces the computational and communication burden of the batteryless node. Instead of transmitting every sample of a high-rate ultrasonic waveform, the embedded system can compute summary quantities, such as RMS, band ratios, peaks, and pulse counts. This creates a hierarchy: high-rate physical sensing at the edge, compact feature representation at the node, and multi-state interpretation in the machine learning layer.

The present analysis uses precomputed window-level feature records. The available implementation does not include the acquisition and window construction procedure, so the exact window duration, observation cadence, stride, and overlap cannot be verified. The ultrasonic sampling rate reported for the earlier hardware characterisation describes waveform acquisition and does not establish the cadence of observations used to construct the machine learning windows. Complete feature formulas, including the conversion of pulse activity into cycling descriptors, are also unavailable. These omissions limit reproducibility and prevent a precise interpretation of cycling-related features in terms of physical cycle frequency or duration.

#### 4.2.2. Feature Taxonomy and Physical Meaning

The feature Table 4 contains several distinct information families. Temperature variables include direct measurements and derived quantities, such as inlet temperature, outlet temperature, ΔT, and absolute ΔT. Acoustic variables include RMS, mean RMS, band ratio, peak value, and peak frequency. Pulse and cycling variables represent the dynamic activity of the trap. Statistical features summarise the distribution within each window using quantities such as mean, standard deviation, minimum, maximum, range, and rolling statistics. Metadata and quality variables describe device context and feature availability.

This taxonomy is important because it allows the ML model to combine variables with different physical roles. Thermal variables describe the state of the process, acoustic variables describe flow-related activity, dynamic variables describe temporal behaviour, and metadata variables describe the measurement context. The model is, therefore, not simply learning a threshold in temperature space—it is learning a nonlinear decision surface across several physical and contextual dimensions.

At the same time, metadata must be handled carefully. Device identifiers and routing variables can be highly predictive of the target without representing physical behaviour. Such variables are precisely why leakage-reduced feature selection is necessary. A high-performing industrial model should rely primarily on variables that remain meaningful when the device population or operating context changes.

### 4.3. Why Multimodal Features Matter

The feature representation combines temperature, ultrasonic/acoustic, and pulse/cycling descriptors because they describe different aspects of observed trap behaviour. Temperature provides thermal context, acoustic measurements describe flow-related activity, and pulse/cycling descriptors represent temporal patterns. This provides a physical rationale for considering the feature families together but does not establish an improvement in classification performance. No temperature-only, ultrasound-only, or cycling-only models were evaluated; consequently, the incremental benefit of the combined representation remains untested. Pulse/cycling descriptors may also be derived from acoustic measurements and should not automatically be regarded as an independent sensing modality.

This is also a central advantage of the proposed IIoT architecture. Because the node is autonomous and continuously connected, the system can build a temporal history rather than relying on a single measurement campaign. That history provides the basis for window-level feature engineering and temporal evaluation.

## 5. Materials and Methods: Machine Learning Methodology

### 5.1. Problem Formulation

The condition-monitoring task is formulated as a supervised six-class classification problem. For each analysis window x, the model estimates a class y belonging to the set {normal, failed-open, failed-closed, degraded, excessive cycling, short cycling}. The objective is not simply to maximise overall accuracy, but to obtain stable class-level behaviour suitable for runtime monitoring.

The distinction between a classification model and a maintenance decision is important. The model predicts an engineering condition category from sensor-derived evidence. It does not directly determine whether a valve should be replaced. This separation reduces the risk of presenting a probabilistic model output as a definitive maintenance diagnosis.

#### 5.1.1. Engineering Labels Versus Ground Truth

The most important methodological qualification in the present study concerns the target labels. The six classes are engineering condition categories generated from sensor-derived operational rules. The dataset description [15] states that they should be interpreted as model output classes rather than direct maintenance work orders, and that final maintenance decisions require field information, device history, and expert inspection, when available.

This distinction changes the interpretation of the reported accuracy. A test accuracy of 98.53% means that the classifier agrees with the evaluation labels for 98.53% of the test windows—it does not mean that 98.53% of field failures have been independently diagnosed correctly. The reported accuracy measures agreement with sensor-derived pseudo-labels, not independently verified diagnostic accuracy. Referring to the task as condition-state classification does not remove the dependence between target construction and model inputs. The classifier may reproduce the operational labelling rules, and the present evaluation cannot distinguish this from recognition of independently confirmed physical faults.

This is not a weakness to conceal. It is a central research design issue and an opportunity for the next experimental phase. Once maintenance records, field inspections, or controlled fault tests become available, the current engineering-label framework can be evaluated against independent outcomes. The resulting experiment would distinguish agreement with the operational taxonomy from true diagnostic validity.

#### 5.1.2. Leakage Risk and Causal Interpretation

Label leakage occurs when a feature contains information that would not legitimately be available at prediction time or directly encodes the rule used to construct the target. In the present dataset, the risk is particularly relevant because the six states are engineering-rule-based. Direct label fields, previous prediction outputs, routing indicators, and high-risk proxy variables can, therefore, create an artificial shortcut for the classifier.

The analysis addresses this issue by defining a non-strict leakage-reduced configuration with 108 features and a stricter configuration with 81 features. The non-strict setting removes direct label-related, prediction-related, routing-related, and high-risk rule-derived variables while preserving informative physical and statistical features. The strict setting removes an additional group of potential proxies.

The resulting performance difference is scientifically informative. If performance was unchanged after aggressive removal, confidence that the model relied on physical information would increase. Instead, macro-F1 decreases substantially under the strict setting. The correct conclusion is, therefore, that the engineered feature space contains valuable information but that some of the retained information may still be closely coupled to the labelling process. Independent labels are required to resolve this ambiguity.

### 5.2. Label Generation and Its Consequences

The available labels are engineering, rule-informed pseudo-labels generated from the combined interpretation of temperature, acoustic, and cycling signals. They are, therefore, not equivalent to independently verified ground-truth failure records. Failed-open and failed-closed patterns are generally more physically interpretable, whereas degraded and cycling-related states are more difficult to separate.

This characteristic creates an important machine learning risk: if the feature set contains variables that directly encode the rules used to generate the labels, a model can achieve very high numerical performance without learning a sufficiently general physical relationship. To address this, the advanced model development stage removes direct label-related, prediction-related, and high-risk rule-derived variables. This is referred to as the leakage-reduced configuration.

However, removing explicit rule indicators and label-related fields does not establish independence between the target labels and the model inputs. Retained temperature, acoustic, and cycling measurements, or their transformations, may still allow the classifier to reconstruct the operational rules used to generate the labels. The reported metrics, therefore, quantify agreement with rule-informed engineering categories rather than independently verified physical fault states. The original numerical labelling thresholds and precedence when rules overlap could not be recovered from the available documentation and implementation files, which limits the reproducibility of the labelling procedure. The available post-processing rules modify model predictions and should not be interpreted as the original label-generation rules.

#### 5.2.1. Why Tree-Based Models

Tree-based models were selected because the input is a structured tabular representation containing nonlinear interactions, mixed scales, and potentially correlated variables. Unlike linear models, tree ensembles can represent threshold-like effects and interactions without requiring an explicit physical equation for every class boundary. They also avoid the need for extensive normalisation when features have different units or distributions.

Random forest [17] provides a strong ensemble baseline through bootstrap sampling and randomised feature selection. Extra trees increases randomisation in the construction of the individual trees [18]. Histogram-based gradient boosting instead builds an additive sequence of trees in which later learners focus on residual classification errors [19]. The latter is particularly suitable when a compact feature representation contains nonlinear interactions that can be refined iteratively.

The comparison is, therefore, not intended as an exhaustive ML benchmark. It is a controlled comparison of three interpretable, deployment-friendly tree approaches that fit the current tabular data representation. More complex deep learning models may become relevant when larger raw time-series datasets are available, but they would also introduce greater requirements for data volume, validation, computational resources, and explainability.

#### 5.2.2. Model Selection Criteria

Model selection is based on four criteria: predictive performance, class-level balance, temporal stability, and deployment suitability. Accuracy is retained because it provides an intuitive overall measure. Macro-F1 is given greater importance because it weights the classes equally and, therefore, reveals whether a model performs poorly on minority states. Temporal stability is assessed using the chronological 70/15/15 split. Finally, the model must operate within the defined current-scope feature schema and support the runtime routing strategy.

This multi-criteria selection is preferable to choosing the model with the highest training accuracy. The training score is particularly uninformative when labels are structured and the model has access to many engineered features. The final model should instead demonstrate that performance remains strong after leakage reduction and when evaluated on later data.

Selection by validation macro-F1 is constrained by validation class coverage. If normal and failed-closed have no reference samples, the selection criterion cannot assess their recovery, although predictions of those classes may still affect the score. A model selected under these conditions is not established as the best-balanced classifier across all six categories. Likewise, evaluation on a later block with incomplete coverage does not validate deployment performance for the absent classes. Registry eligibility provides no substitute for class-specific validation.

### 5.3. Candidate Models

Three tree-based classifiers are evaluated in Table 5: random forest, extremely randomised trees, and histogram-based gradient boosting. Tree ensembles are appropriate for the structured tabular feature representation because they can model nonlinear interactions without requiring the signal distributions to be transformed into a linear form.

Random forest constructs an ensemble of decision trees using bootstrap sampling and feature randomisation. Extremely randomised trees increase randomisation in the tree construction process. Histogram-based gradient boosting builds an additive sequence of trees, where later trees focus on errors made by previous trees. The latter can efficiently model nonlinear interactions in a relatively high-dimensional tabular feature space.

The recovered training script specifies fixed hyperparameters for each candidate. Random forest uses 600 trees, maximum depth 12, minimum leaf size 8, minimum split size 12, and balanced_subsample class weighting. Extra trees uses 800 trees, maximum depth 14, minimum leaf size 6, minimum split size 10, and balanced class weighting. Histogram-based gradient boosting uses 300 maximum iterations, learning rate 0.05, 31 maximum leaf nodes, and L2 regularisation of 0.1, with balanced sample weights calculated from the training labels. All three candidates use random_state = 42, and unspecified parameters retain the library defaults.

The script contains no grid search, randomised search, or other automated hyperparameter optimisation. It fits each candidate on the training subset and selects the model with the highest validation macro-F1, using validation accuracy to resolve ties. Test performance is calculated for each candidate but is not used by the selection function. These settings describe the recovered implementation—their correspondence to the original runs producing the published scores could not be independently verified.

For a given class, precision measures the fraction of predicted positives that are correct, while recall measures the fraction of actual class instances that are recovered. The F1-score combines both quantities. Macro-F1 is calculated as the unweighted mean of the class-level F1 scores. This makes it particularly relevant when some states occur much less frequently than others.

The distinction between weighted and macro metrics is central to this application. A failed-open or degraded state may dominate the available windows, while excessive cycling and short cycling may be comparatively rare. A classifier could, therefore, obtain high accuracy while almost ignoring one of the minority classes. Macro-F1 prevents the majority classes from completely masking this behaviour.

Confusion matrices are equally important because they identify physically meaningful error boundaries. Confusion between failed-open and failed-closed would be operationally significant. Confusion between excessive cycling and short cycling is different: both represent dynamic abnormalities and may have overlapping physical signatures. The final interpretation, therefore, combines aggregate scores with the engineering meaning of each error.

### 5.4. Evaluation Metrics

Accuracy is calculated as the fraction of correctly classified windows. Because the six classes are not equally represented, accuracy alone is insufficient. Precision, recall, and F1-score are, therefore, calculated at class level. For a class, precision is TP/(TP + FP), recall is TP/(TP + FN), and F1 is the harmonic mean of precision and recall. Macro-F1 is an unweighted mean of class-level F1 scores. In the recovered script, the scalar calculation includes the classes present in the reference labels or predictions, whereas the classification report explicitly includes all six categories. These averages may differ when categories are absent—the exact calculation underlying the published scores remains unverified.

Confusion matrices are used to identify which states are systematically confused. This is particularly relevant for degraded, excessive-cycling, and short-cycling states, where the physical boundaries may be less distinct than for failed-open or failed-closed conditions.

The available evaluation procedure assigns records to 70/15/15 blocks after sorting by series or device and then by timestamp. As detailed in Section 5.5, this does not guarantee global chronological separation, device-disjoint subsets, or independence between adjacent windows. The results should, therefore, be interpreted within those limitations.

The available study description reports that normal and failed-closed are absent from the later validation and test blocks. Their recall cannot be assessed in those periods, and the experiment, therefore, does not provide complete six-class temporal validation. In the recovered script, scalar macro-F1 averages over the union of classes appearing in the reference labels and predictions, whereas the classification report explicitly includes all six classes with zero_division = 0. Consequently, the two averages can differ. The scalar average may also include an absent reference class if the model predicts it, meaning that comparisons between models or feature configurations may involve different class sets. The published scores cannot be interpreted as a verified fixed six-class average without the original run outputs.

Reference-label counts for each class in the training, validation, and test subsets underlying the reported scores are unavailable in the recovered run materials. The reported absence of classes could, therefore, not be independently verified. Runtime prediction counts cannot substitute for reference-label distributions within the evaluation subsets. This missing information limits interpretation of class imbalance, class coverage, and the reported macro-F1 values.

No repeated-seed, repeated-partition, or resampling analysis is available, and confidence intervals are not reported. The aggregate scores alone do not support a reliable uncertainty estimate or a paired statistical comparison between models. Treating individual windows as independent resampling units could underestimate uncertainty because neighbouring records may share observations or operating conditions. An appropriate analysis would require reference labels and predictions for each model on the same evaluation records, together with justified temporal blocks or operating-episode groups. The reported differences are, therefore, descriptive and do not establish statistical superiority.

Random forest and extra trees serve as alternative algorithmic comparators, but no majority-class, simple physical threshold, or original-rule baseline was evaluated. In particular, agreement with rule-generated targets should not be interpreted as improvement over the generating rules without independent reference labels.

### 5.5. Temporal Evaluation

Random splits can produce optimistic estimates when neighbouring windows from the same device or operating episode appear in both training and test sets. The available evaluation script instead sorts records by series identifier, or device identifier when the series identifier is unavailable, and then by timestamp within each group. It assigns the first 70% of the resulting concatenated records to training, the next 15% to validation, and the remaining 15% to testing. This procedure preserves temporal ordering within each series or device but does not guarantee a global chronological separation or device-disjoint subsets. Consequently, it should not be interpreted as an unseen-device evaluation.

The partition is applied to the concatenated records rather than independently within each device, so device representation may differ between subsets, and the same device may contribute to more than one subset. Operating episodes are not used as grouping units, and no exclusion gap is introduced at subset boundaries. Since window duration and overlap are undocumented, it cannot be established whether adjacent windows share raw observations or belong to the same operating episode. Even non-overlapping windows may remain correlated when operating conditions persist. The reported split, therefore, does not establish independence between training and evaluation samples, and its scores may be optimistic.

The exact device and episode membership of the run that produced the published metrics could not be verified from the available aggregate results. For 6354 input rows, the recovered script assigns 4447 records to training, 953 to validation, and 954 to testing, whereas the manuscript previously listed 4449, 954, and 951. These counts cannot be treated as interchangeable without the original run records.

The purpose of the time-block evaluation is not only to obtain a second accuracy number. It tests whether the learned relationship remains stable when the model is exposed to later observations. Changes in operating conditions, device behaviour, maintenance events, and sensor quality can produce temporal drift. A model that performs well only under a random split may, therefore, be unsuitable for continuous industrial deployment.

Scope-aware routing implements a predefined device eligibility policy. A registry indexed by dataset and device identifier assigns records to current-scope, legacy, or out-of-scope categories. This assignment restricts formal predictions to designated device series but does not test whether incoming sensor measurements follow the training distribution. Changes in operating conditions or sensor behaviour within a registered current-scope device are, therefore, not detected by the routing mechanism.

The runtime architecture distinguishes three paths. Current-scope records proceed to formal six-state predictions. Legacy records can be processed through shadow inference for exploratory comparison, but their outputs are not treated as formal diagnoses. Out-of-scope records are rejected from formal predictions. The runtime evaluation demonstrates this routing on 23,684 window-level records, of which only 6354 were in the current scope.

This conservative design is especially appropriate for industrial AI because a system should be able to say ‘unsupported’ rather than generate a confident output outside the distribution for which it was evaluated. In a production system, this mechanism could be extended with automated drift detection, feature-schema monitoring, and model-version qualification.

The temporal evaluation does not establish generalisation to unseen devices. The available partitioning procedure does not enforce device-disjoint subsets, and records from the same monitoring device may occur in both training and evaluation sets. No leave-one-device-out or cross-facility evaluation was performed. Consequently, the reported scores should be interpreted within the evaluated dataset and should not be extrapolated to new monitoring devices, steam traps, or industrial facilities.

### 5.6. Scope-Aware Runtime Strategy

The runtime pipeline separates devices into three groups. Current-scope devices have the feature structure and signal behaviour used for formal model development. Legacy devices can be processed through shadow inference for exploratory analysis but do not receive formal six-state outputs. Out-of-scope devices are rejected from formal diagnosis.

This mechanism is deliberately conservative. A machine learning model should not be applied to an input distribution for which its training data provide no evidence of validity. In industrial monitoring, a transparent ‘not supported’ output is preferable to a confident but unsupported fault classification.

### 5.7. Post-Processing and Runtime Interpretation

The runtime workflow can apply conservative post-processing after the base model prediction. The purpose is to avoid over-interpreting ambiguous predictions and to maintain a clear distinction between model output and maintenance action. The dashboard representation includes prediction status, device scope, and supporting metadata, allowing operators to inspect the context of each output.

### 5.8. Software, Reproducibility, and Deployment Controls

The machine learning pipeline was implemented in Python 3.13.5 using scikit-learn 1.8.0 [20]. Numerical missing values were imputed using the median, categorical missing values using the most frequent category, and categorical variables were ordinal-encoded with unseen categories assigned a value of −1. A random state of 42 was used for model training. The same preprocessing pipeline and feature schema were maintained across training, validation, testing, and runtime inference. The implementation code, trained-model package, scope-routing configuration, post-processing rules, and dashboard are publicly available at: https://github.com/1822971148-ux/steam-trap-predictive-maintenance (accessed on 10 July 2026).

### 5.9. Feature-Group Interpretation and Error Analysis

The classifier should be interpreted as a multimodal model rather than an acoustic-only or temperature-only detector. Thermal features provide slow process context, ultrasonic features provide sensitivity to flow and leakage dynamics, and pulse/cycling features describe opening and closing behaviour. The engineered representation, therefore, converts high-rate physical signals into compact variables with direct engineering meaning.

## 6. Results

Results reported in this section concern classifier comparison, feature-set sensitivity, and runtime demonstration using the available window-level records. The thermoelectric measurements summarised in Section 3 were reported previously [7] and are not experimental results generated for the present analytical study. No new whole-node energy-budget experiment or independently confirmed fault-validation campaign is reported here.

The monitoring records analysed in this study were collected at a pharmaceutical facility in Barcelona between August 2025 and March 2026. Figure 5 shows the installation arrangement.

The numerical comparison shows a clear separation between the candidate models. Random forest reaches 0.8030 validation macro-F1 and 0.7267 test macro-F1, while extra trees reaches 0.7171 and 0.4810, respectively. Histogram-based gradient boosting reaches 0.9550 validation macro-F1 and 0.8822 test macro-F1. The same ordering is observed in accuracy, with HistGradientBoosting achieving 0.9958 validation accuracy and 0.9853 test accuracy.

The difference in macro-F1 is more important than the difference in accuracy. Extra trees, for example, retains a relatively high overall test accuracy of 0.9403, while its test macro-F1 falls to 0.4810. This indicates that the model can classify a substantial number of majority-class windows correctly while failing to maintain balanced performance across the six-state taxonomy. HistGradientBoosting shows a much smaller gap between accuracy and macro-F1 and is, therefore, the stronger candidate for a condition-monitoring application.

The result also provides a practical reason for preferring gradient boosting over the other two models in the current dataset. The feature representation contains nonlinear combinations of thermal, acoustic, and dynamic variables. Sequential boosting can refine decision boundaries around difficult regions, whereas the randomised tree ensembles appear less effective at preserving minority-class performance.

### 6.1. Model Comparison

Histogram-based gradient boosting obtained the highest reported validation and test macro-F1 among the three candidates under the leakage-reduced configuration—statistical significance of these differences was not assessed. Its validation macro-F1 of 0.9550 was substantially higher than random forest at 0.8030 and extra trees at 0.7171. The difference was also maintained in the chronological test period, where the three models achieved macro-F1 values of 0.8822, 0.7267, and 0.4810, respectively (Figure 6).

The result is important because macro-F1 penalises models that perform well on dominant classes but poorly on minority classes. For a steam-trap monitoring application, correct classification of a less frequent abnormal state can be operationally more valuable than achieving a marginal increase in overall accuracy on the majority class.

The time-block results show a gradual reduction from the training period to the later test period: accuracy changes from 1.0000 to 0.9958 and then 0.9853, while macro-F1 changes from 1.0000 to 0.9550 and then 0.8822. The decline is expected when the model is evaluated on later observations, but the absolute test performance remains high for the classes represented in the test block. Perfect agreement on the training block may reflect overfitting or shared information between engineering labels and model features—it does not establish physical fault-diagnosis accuracy.

The chronological test should, therefore, be interpreted as evidence of useful temporal stability rather than proof of universal generalisation. The absence of normal and failed-closed from the later blocks means that those two classes are not being challenged under the same temporal conditions. A future dataset containing long, continuous records from all six states would allow a much stronger temporal experiment.

### 6.2. Time-Block Performance

The reported aggregate scores show lower macro-F1 in the final evaluation block than in validation (Figure 7 and Table 6) but do not identify which classes account for that decrease. Although the study description reports greater difficulty with cycling-related categories, the original class-level reports and confusion matrices are unavailable for verification. Consequently, class-specific precision, recall, and F1, and the contribution of individual confusion patterns, cannot be established from the recovered results. Categories without reference samples in an evaluation subset cannot have their recall assessed in that subset.

An additional limitation of the temporal experiment is that the later blocks do not contain all six operating states. In particular, normal and failed-closed samples are absent from the later chronological blocks. Consequently, the temporal results demonstrate stability for the classes represented in those periods but cannot be interpreted as equal temporal validation of all six states.

Section 6.3 reports the comparison between the two feature-exclusion configurations and discusses its interpretation.

### 6.3. Robustness and Leakage Reduction

The reported analysis compares a non-strict leakage-reduced configuration containing 108 features with a stricter 81-feature configuration. Validation macro-F1 decreases from 0.9550 to 0.6307, and test macro-F1 from 0.8822 to 0.4999 (Figure 8).

The strict configuration excludes additional temperature difference, pulse/cycling, and ratio descriptors, removing potentially useful physical information alongside possible proxies for the labelling rules. The performance decrease demonstrates sensitivity to the excluded feature set but does not distinguish loss of useful sensor information from reduced access to label-generation proxies. Since the additional features were removed jointly and no individual or grouped ablation was performed, their specific contributions cannot be established. This comparison also does not isolate sensing modalities and, therefore, does not establish an advantage of combined inputs over single-modality models.

No complete rule-to-feature audit or device-disjoint evaluation was performed. Furthermore, the exact class averaging and partition underlying the published scores remain unverified. The comparison should, therefore, be interpreted as a feature-set sensitivity analysis, rather than evidence that dependence on the labelling rules has been eliminated or that the retained features are independently validated physical predictors.

### 6.4. Runtime Scope Distribution

The runtime demonstration assigned 23,684 window-level records to predefined scope categories, shown in Table 7. Of these, 6354 were designated current scope, 4511 legacy, and 12,819 out of scope, corresponding to 26.8%, 19.0%, and 54.1%, respectively (Figure 9). These counts describe application of the registry-based policy and do not measure distribution-shift detection performance.

The registry separates records eligible for formal prediction from legacy shadow records and rejected records. This demonstrates application of the predefined device-scope policy but does not establish statistical compatibility or diagnostic validity for accepted inputs. Section 6.5 presents the distribution of predicted engineering categories within the current-scope records.

### 6.5. Formal Six-State Distribution

Figure 10 and Table 8 summarise the predicted engineering categories for the 6354 current-scope records. These counts describe model outputs, not independently confirmed physical faults or plant-level failure prevalence.

### 6.6. Runtime Dashboard and Device-Level Visualisation

The runtime dashboard provides the final human-facing layer of the proposed monitoring architecture. Rather than presenting the machine learning output as an isolated class label, the prototype exposes the data-loading status, model scope, prediction status, and device-level information required to interpret the result. The prototype processes the scope-aware runtime output and distinguishes formal current-scope predictions from legacy shadow inference and rejected out-of-scope records (Figure 11).

The overview screen gives an immediate indication of whether the runtime dataset is being processed as expected. It reports the total number of windows together with the number receiving formal predictions, legacy shadow outputs, and rejected out-of-scope records. In the demonstrated run, 23,684 windows were processed, of which 6354 were current-scope records, 4511 were legacy, and 12,819 were out of scope (Figure 12). This view is useful from an industrial operations perspective because it makes model qualification visible before individual predictions are interpreted.

The Device Explorer moves from fleet-level monitoring to asset-level inspection. For a selected device, the prototype displays the number of available windows, model and scope status, mean prediction confidence, and the distribution of the six condition states. This is particularly valuable for maintenance-oriented interpretation because a single instantaneous classification can be misleading, whereas the distribution across many windows reveals whether an asset is predominantly normal, degraded, failed, or cycling (Figure 13). This view provides a device-level inspection function.

The time-series view provides the missing link between the numerical prediction and the underlying physical measurements. By displaying the selected features over time, the operator can inspect whether a predicted condition is accompanied by a persistent thermal or acoustic signature, a transition, or a cycling pattern. This is important for explainability: the dashboard does not claim to provide causal explanations, but it allows the user to inspect the sensor evidence associated with a prediction rather than treating the classifier as a black box.

Taken together, the three views demonstrate an end-to-end workflow from runtime data ingestion to fleet-level scope control, asset-level condition distribution, and time-series inspection. The dashboard remains a prototype rather than a production maintenance platform: the prototype description notes that it does not yet integrate live industrial streams, automated work orders, or long-term operator feedback.

Nevertheless, including the visualisation layer is important because it shows how the ML output could be incorporated into an industrial decision-support workflow.

### 6.7. What the Results Demonstrate and What They Do Not

The results demonstrate that a multimodal, window-based feature representation can support high-performing six-state classification within a defined device scope. They also demonstrate that the selected HistGradientBoosting model remains strong under chronological evaluation and that a conservative routing mechanism can prevent unsupported records from being treated as formal predictions.

The results do not yet demonstrate universal steam-trap fault diagnosis. The labels are engineering-rule-based, the later time blocks do not contain all six states, and the device population is limited. These constraints are not secondary details—they define the scientific boundary of the result. The most defensible claim is, therefore, that the proposed workflow provides a promising and technically coherent condition-monitoring prototype for supported devices.

## 7. Discussion

The reported results should be interpreted in relation to differences in sensing, reference labels, and evaluation design, rather than compared through accuracy alone, shown in Table 9. Kim et al. [1] address steam-trap diagnosis using statistical time-series features and a Transformer encoder–decoder, with data from aluminium-processing and food-manufacturing facilities. Their labelling involved diagnostic engineers and disassembly of selected suspect traps. The present analysis instead evaluates precomputed thermal, acoustic, and pulse/cycling descriptors against sensor rule pseudo-labels from a restricted monitoring-device population. This difference in reference evidence prevents interpreting a higher numerical score as a better physical diagnosis.

Li et al. [2] address acoustic-emission denoising, evaluating signal reconstruction through signal-to-noise ratio and root-mean-square error. Their results concern the quality of acoustic evidence, not agreement with a six-category taxonomy. The present study does not independently evaluate noise robustness. Likewise, thermoelectric sensing [4] and energy-aware LoRaWAN communication [5] provide relevant implementation comparisons, but their energy results cannot establish autonomy of a different workload.

The following comparison separates these evaluation objectives. No common dataset, harmonised trap population, or shared labelling protocol was used; consequently, it is a comparison of evidence and scope rather than a performance ranking.

This comparison identifies the principal boundary of the present contribution. The reported scores support analysis of agreement with an engineering taxonomy, while the feature-exclusion experiment exposes sensitivity that requires further investigation. They do not establish superiority over field-supported diagnostic methods, robustness across trap technologies or operating conditions, or operational maintenance benefits.

### 7.1. From Leak Detection to Condition Monitoring

The principal advance over the previous steam-trap IIoT work is the change in the information produced by the system. The earlier device demonstrated that thermoelectric energy harvesting, temperature, and ultrasound can support autonomous steam-leak monitoring. The present work uses the same physical sensing foundation but introduces temporal feature engineering and supervised classification to produce a richer condition description [7].

This transition is significant for industrial maintenance. A binary alarm can identify a problem, but a multi-state condition indicator can support prioritisation. A failed-open state is associated with direct steam loss and, therefore, energy and emissions impact. A failed-closed state can indicate condensate management risk. Degraded and abnormal cycling states may provide earlier indications of developing problems. The model, therefore, becomes an interpretation layer between raw sensor measurements and maintenance workflow.

### 7.2. Why Thermoelectric Powering Matters to Machine Learning

The relationship between energy harvesting and machine learning is indirect but fundamental. Continuous monitoring requires a sustainable energy budget. If the sensor depends on a battery with a limited service interval, acquisition and communication frequency must be constrained, or maintenance must periodically replace the energy source. A thermoelectric node instead uses the process heat that is already available at the installation point.

Thermoelectric powering provides a rationale for repeated sensing while a sufficient thermal gradient is available. However, the present study does not quantify whether harvested energy sustains the proposed acquisition and communication schedule across field conditions. Its contribution is the analysis of the resulting window-level data—verification of the complete node energy budget remains necessary.

### 7.3. Industrial Scalability

The reported performance describes agreement with engineering labels within a dataset from two monitoring-device series at one pharmaceutical facility. It does not estimate the probability of correctly diagnosing a physical fault on a new trap or at another site. Likewise, window-level accuracy cannot be translated directly into an operational false-alarm rate, missed-fault rate, or maintenance benefit: successive windows may be correlated, and their predictions have not been linked to independently confirmed fault episodes or intervention outcomes.

The practical contribution is a prototype that organises sensor-derived records into engineering categories and makes prediction scope and sensor context visible for review. Its operational usefulness remains to be established. Broader applicability would require device-disjoint and cross-site evaluation with independent reference labels, while industrial scalability would additionally require assessment of communication capacity, energy availability, installation effort, and maintenance system integration. The registry limits formal output to designated series—it does not demonstrate transferability or continued validity when operating conditions change.

### 7.4. Environmental Impact

Potential environmental benefits must be distinguished from outcomes measured in this study. No maintenance intervention, avoided steam loss, fuel saving, or emissions reduction was measured. The previously published leakage example is illustrative and should not be interpreted as a saving achieved by the present workflow [7].

A site-specific assessment would require confirmed leakage rates, operating duration, and intervention outcomes. For avoided steam mass M, the corresponding avoided fuel energy can be approximated as E_fuel = M(h_steam − h_makeup)/η_boiler, using consistent units and documented assumptions. Avoided emissions can then be estimated from E_fuel and an emission factor expressed per unit of fuel energy. Pressure, replacement water conditions, boiler efficiency, and fuel type must be specified—a universal emissions saving cannot be inferred from classification scores.

Any reduction in battery-related environmental burden would require a separate lifecycle comparison accounting for harvesting hardware, storage, installation, and maintenance, according with Table 10. Both process savings and monitoring system impacts remain subjects for future measurement rather than demonstrated benefits of this study.

### 7.5. Limitations

The central validity limitation is the dependence between the engineering labels and the sensor-derived model inputs. The reference categories were constructed from thermal, acoustic, and pulse/cycling information, and the classifier receives descriptors from these same signal families. High agreement may, therefore, reflect reconstruction of the labelling rules rather than recognition of independently confirmed physical faults. Neither temporal partitioning nor removal of explicit rule indicators establishes diagnostic validity.

The reported metrics quantify agreement with the engineered taxonomy. The decrease under stricter feature exclusion reinforces the need to examine label–feature dependence but does not isolate its cause because potentially useful physical descriptors were removed simultaneously. Independent inspections, maintenance outcomes, or controlled fault experiments are required to assess physical diagnostic validity. Additional limitations include the restricted device population, incomplete acquisition documentation, unverified class coverage of the reported evaluation, and possible dependence between training and evaluation windows.

Several mechanisms may affect performance during deployment. Sensor drift can change measured levels without a corresponding change in trap condition, while acoustic interference and variations in sensor mounting or thermal contact can alter the recorded descriptors. Trap technology and operating conditions can also change the relationship between these descriptors and physical behaviour. These effects were not separately quantified in the present study. A change in input distributions would not necessarily imply a fault, and the registry-based routing mechanism would not detect such a change in an already eligible device.

Concept drift concerns changes in the relationship between observations and the condition of interest, for example, following changes in process operation or maintenance. Monitoring agreement with unchanged sensor-derived rules would not establish that this physical relationship remains valid. Detecting and assessing such changes requires contextual records and independent inspection outcomes.

Operational errors also have different consequences. A false failed-open indication could prompt unnecessary inspection or replacement, whereas a missed failed-open condition could delay intervention on continuing steam loss. A false failed-closed indication could prompt unnecessary investigation of condensate discharge, while a missed failed-closed condition could leave insufficient discharge unaddressed. Confusion between the two could direct attention toward an inappropriate failure mechanism. These are potential consequences, not outcomes observed in this study. False-alarm and missed-fault rates require independently labelled events, and correlated window predictions should not be counted as separate maintenance incidents. Until such validation is available, outputs should support inspection rather than independently determine maintenance action.

Further, although the model achieves high numerical performance, field validation under confirmed fault conditions remains necessary. Finally, the present work evaluates the ML pipeline primarily at the window level. Future studies should connect predictions to maintenance records, intervention outcomes, and measured steam-loss reductions.

## 8. Deployment Roadmap and Future Experimental Validation

Independent fault validation and cross-device testing are prerequisites for claims of physical diagnostic accuracy and generalisation beyond the evaluated device population, respectively. Their inclusion in this roadmap does not substantiate such claims in the present manuscript. The current evidence is restricted to classification agreement with rule-informed labels and demonstration of a runtime prototype within a predefined scope. The validation programme below describes the evidence required before stronger diagnostic and deployment claims can be made.

A prospective validation programme should separate physical diagnostic validity, generalisation, and operational reliability. The following protocol is proposed for future work—it was not performed in the present study. Numerical acceptance thresholds and sample sizes must be specified before data collection, using site requirements and the desired statistical precision rather than selected after observing results.

The sampling frame should identify individual physical traps, monitoring devices, trap technology, application, and installation. Mechanical, thermodynamic, and thermostatic traps should be represented where they occur in the intended deployment population. Operating pressure, condensate load, backpressure, thermal conditions, and relevant acoustic interference should be recorded. Evaluation should include multiple devices and facilities, with devices and fault episodes kept separate between model development and final testing.

Independent reference labels should be assigned through documented inspection by qualified personnel, supported where feasible by controlled fault tests, disassembly, or maintenance findings. Assessors should be blinded to classifier outputs, and disagreements and uncertain cases should be recorded. Post-repair observations can provide supporting evidence but should not alone determine the reference state. The proposed degraded and cycling categories require reproducible physical definitions and assessment of agreement between assessors before six-state diagnostic validation is claimed.

Sample-size planning should use independently confirmed episodes and physical traps as the principal units, rather than treating successive windows as independent observations. For each category, the required number of reference episodes should be calculated to achieve a prespecified confidence interval width for sensitivity. Confirmed non-fault operating exposure is additionally required to estimate false-alert rates. Repeated episodes from the same trap must be accounted for through grouped analysis. If sufficient evidence cannot be obtained for a category, its validation should remain explicitly incomplete.

Monitoring duration should cover the operating envelope claimed for deployment, including relevant load changes, maintenance transitions, low thermal gradients, and restart conditions. Seasonal coverage is required if year-round performance is claimed. A minimum calendar duration and minimum normal-operation exposure should be specified in the protocol, and observation should also continue until the required class-specific event counts are obtained. Duration alone cannot compensate for missing fault categories.

Diagnostic acceptance criteria should include prespecified lower confidence bounds for class-specific sensitivity and precision, an upper bound for false alerts per trap-day, and a maximum acceptable detection delay. Failed-open and failed-closed errors should be reported separately because their operational consequences differ. Macro-F1 should use a fixed, explicitly stated class set, with reference support and absent classes reported. Performance should be examined by trap technology, operating condition, device, and site, using dependence-aware confidence intervals.

Operational acceptance should additionally require documented data completeness, packet-delivery performance, and energy balance under the intended acquisition and communication schedule. Cold-start time, interruption recovery, and storage-supported autonomy should be measured. Results should be compared with a simple reference method and the original operational rules using the same independent labels. Formal deployment should be limited to categories and conditions that meet the predefined criteria—unmet criteria and unsupported conditions should remain explicit.

Industrial deployment should maintain a versioned record of the model, preprocessing pipeline, feature schema, device registry, and post-processing rules. Each prediction should retain the applicable versions, device identifier, timestamp, and input-quality status. Monitoring should track missing data, implausible values, prediction distributions, and independently confirmed diagnostic errors. Sensor calibration checks should follow documented instrument and site procedures. Model updates should require reviewed reference data, separate validation, and approval before release, with a recoverable previous version for rollback.

Distribution monitoring should compare incoming features with documented reference distributions using thresholds established during validation. Drift alerts should trigger investigation of sensor condition, installation, and process changes—they should not automatically trigger retraining or be interpreted as confirmed faults. Communication failures should produce an explicit unavailable or stale-data status, with timestamps, gap logging, and defined buffering and recovery procedures. Security controls should include authenticated device access, protected communications, least-privilege permissions, controlled software updates, and audit logs. Integration with a maintenance management system should link alerts to asset identifiers, inspection records, and intervention outcomes, with human review before issuing work orders. These controls are proposed deployment requirements and were not implemented or evaluated as a complete industrial system in this study.

## 9. Conclusions

This study examined a window-level analytical workflow built on a previously reported thermoelectrically powered steam-trap sensing platform. The six target categories are sensor-derived, rule-informed pseudo-labels rather than independent physical ground truth. The reported histogram-based gradient boosting test accuracy of 0.9853 and macro-F1 of 0.8822, therefore, measure agreement with those labels, not independently validated fault-diagnosis accuracy. Under stricter feature exclusion, test macro-F1 decreases to 0.4999. This substantial reduction indicates sensitivity to the feature representation but does not isolate dependence on labelling rules from loss of useful sensor information.

The available study description reports that normal and failed-closed are absent from the later validation and test blocks; consequently, the evaluation does not validate all six classes over time. Original class counts and evaluation outputs are unavailable for independent verification. Label–feature dependence, incomplete class coverage, and possible correlation between windows restrict the interpretation of the reported scores. Generalisation to unseen devices or facilities, statistical superiority, and an advantage over single-modality models remain unestablished.

The runtime prototype demonstrates application of a predefined device registry and presentation of prediction status and sensor context. It does not demonstrate distribution-shift detection, sustained autonomy of the complete sensing workload, or measured maintenance and environmental benefits. Independent physical labels, reproducible acquisition and evaluation records, and device-disjoint testing are required before stronger diagnostic or deployment claims can be supported.

## Figures and Tables

**Figure 1:**
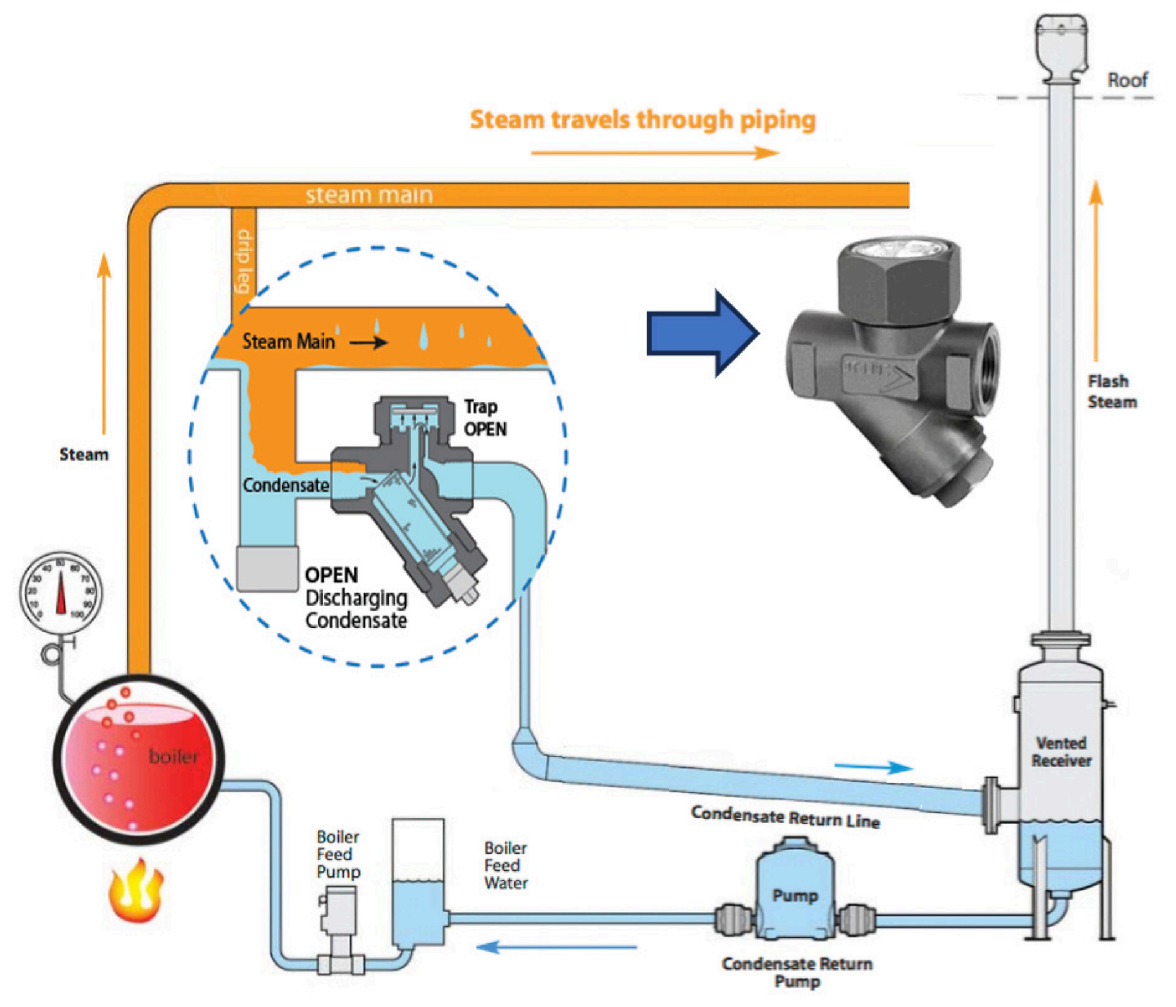
Steam circuit composed of the boiler, steam pipeline, and steam trap.

**Figure 2:**
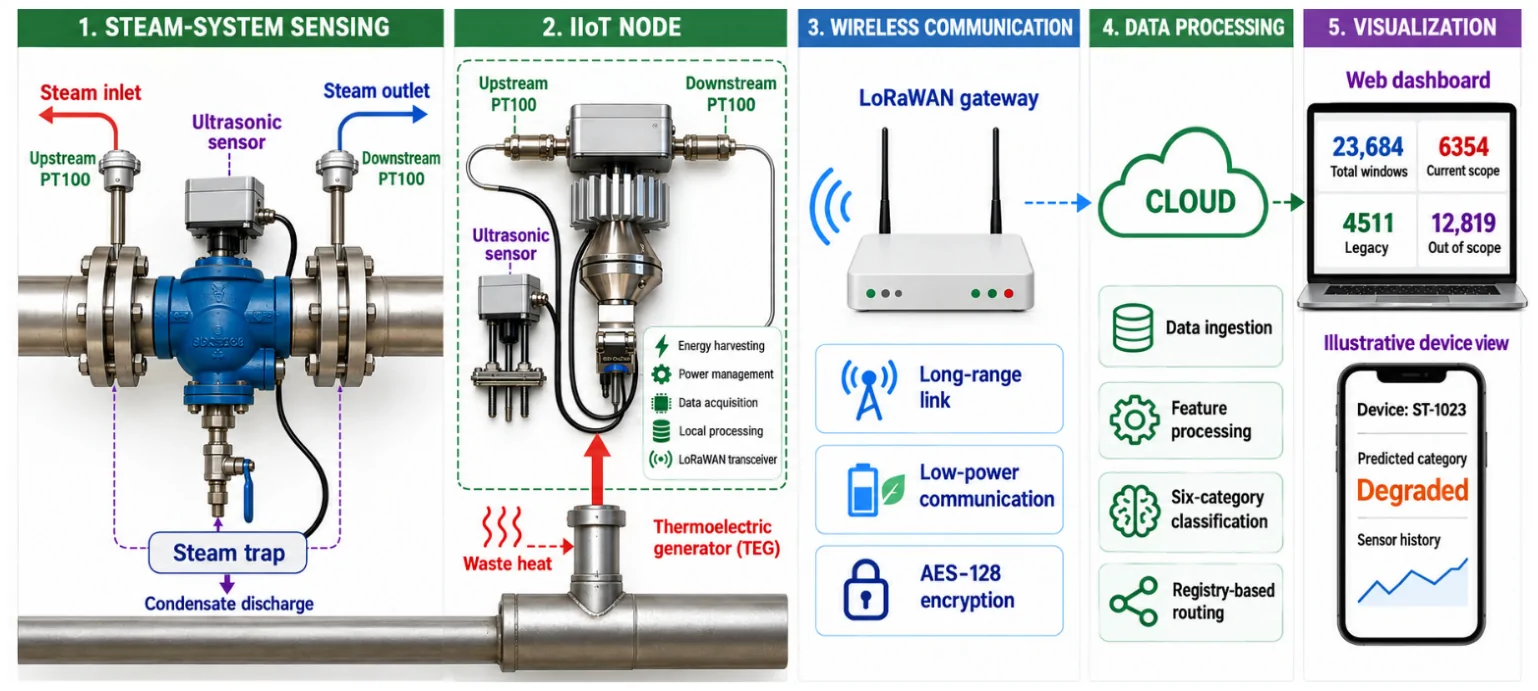
IIoT architecture for steam-trap condition monitoring.

**Figure 3:**
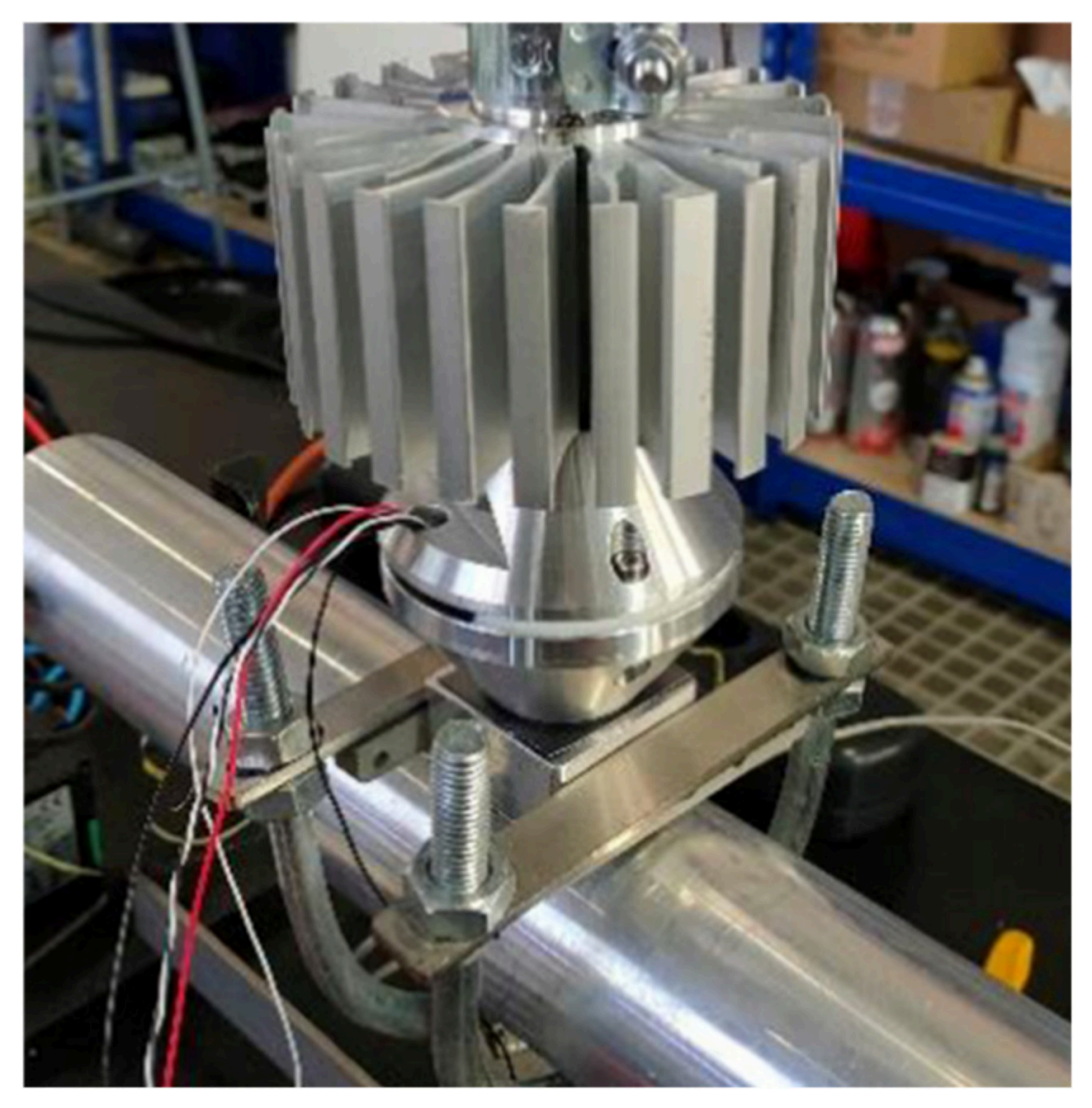
Previously reported thermoelectric characterisation setup [7].

**Figure 4:**
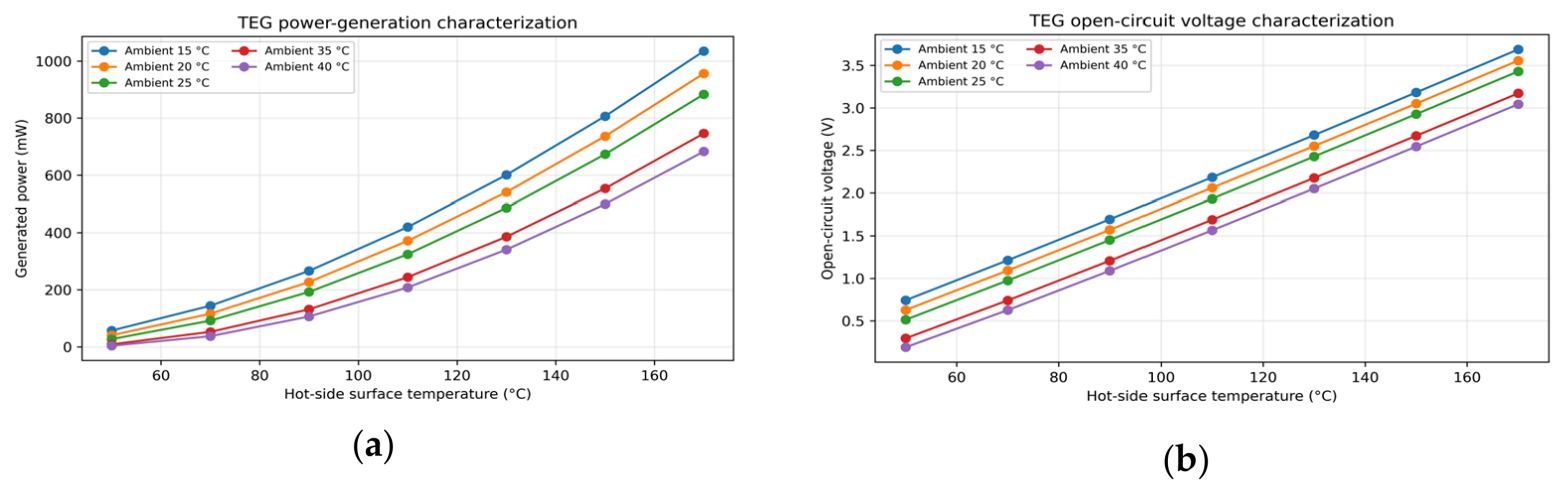
(**a**) Generated electrical power of the thermoelectric generator as a function of hot-side surface temperature for the ambient temperatures reported in the previous study. Data reproduced from the original experimental dataset. (**b**) Open-circuit voltage of the thermoelectric generator as a function of hot-side surface temperature for the ambient temperatures reported in the previous study. Data reproduced from the original experimental dataset. Previously reported measurements from [7]—no new thermoelectric measurements are presented here.

**Figure 5:**
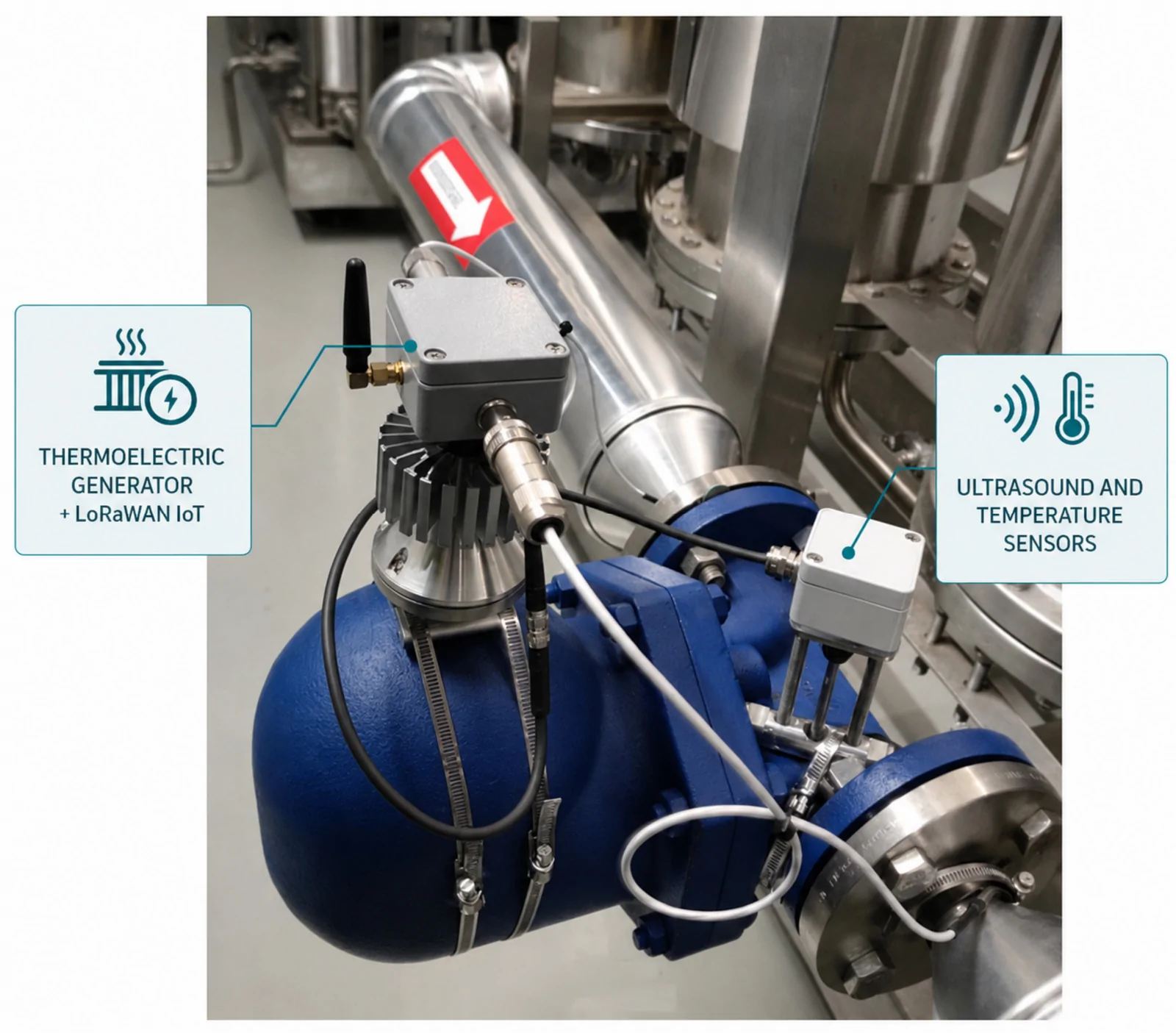
System implementation in a pharma facility on a mechanical steam trap.

**Figure 6:**
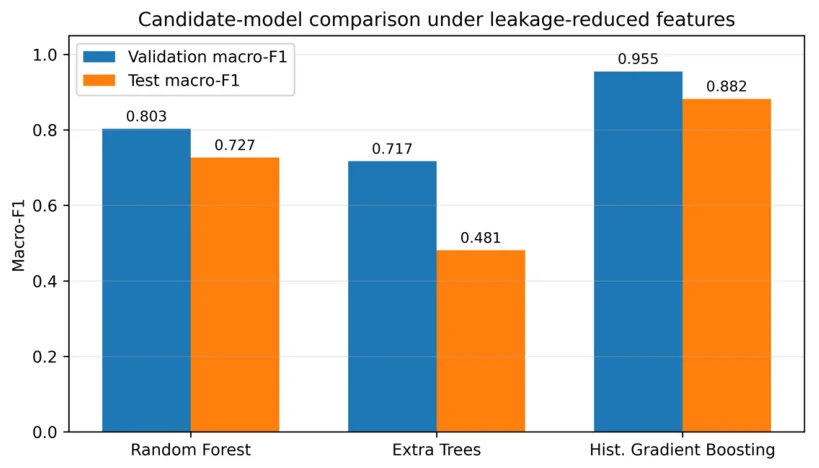
Validation and test macro-F1 for the three candidate classifiers under the non-strict leakage-reduced configuration.

**Figure 7:**
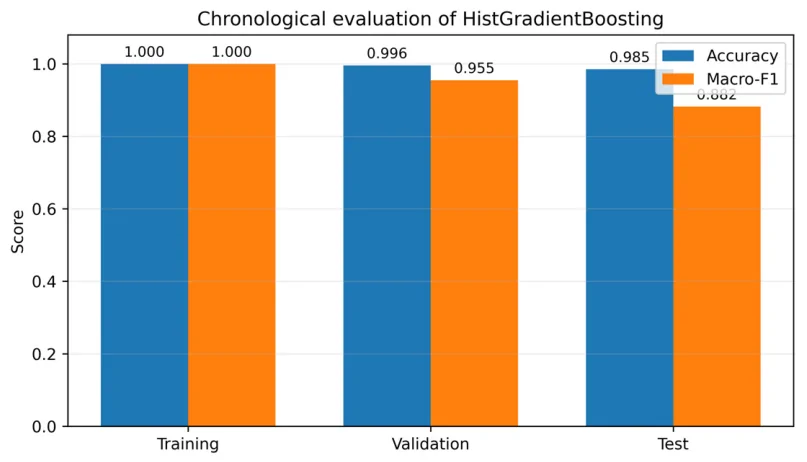
Accuracy and macro-F1 across the chronological training, validation, and test blocks for the selected histogram-based gradient boosting model.

**Figure 8:**
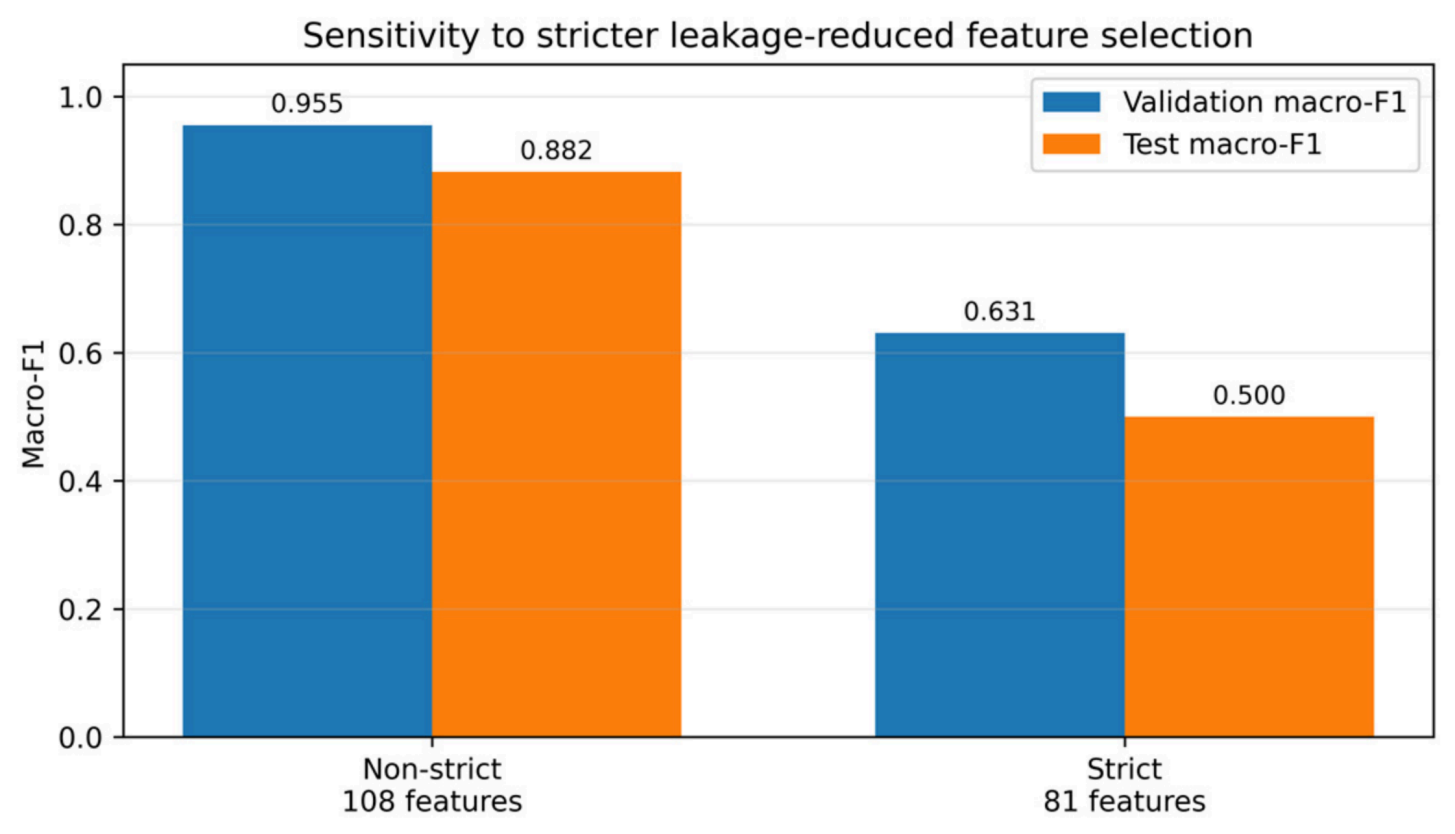
Reported validation and test macro-F1 under non-strict and strict feature-exclusion configurations.

**Figure 9:**
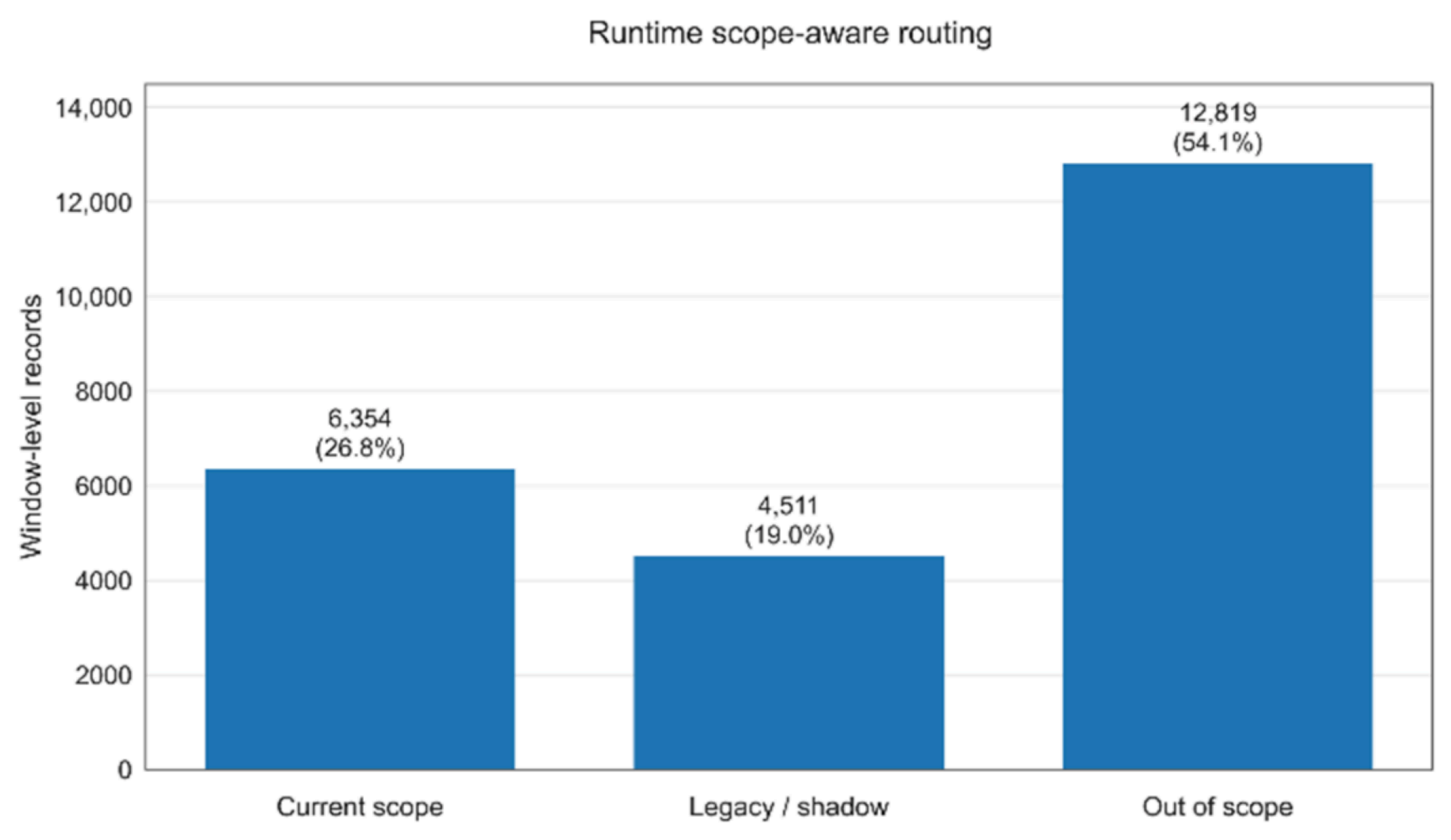
Runtime distribution of current-scope, legacy/shadow, and out-of-scope records.

**Figure 10:**
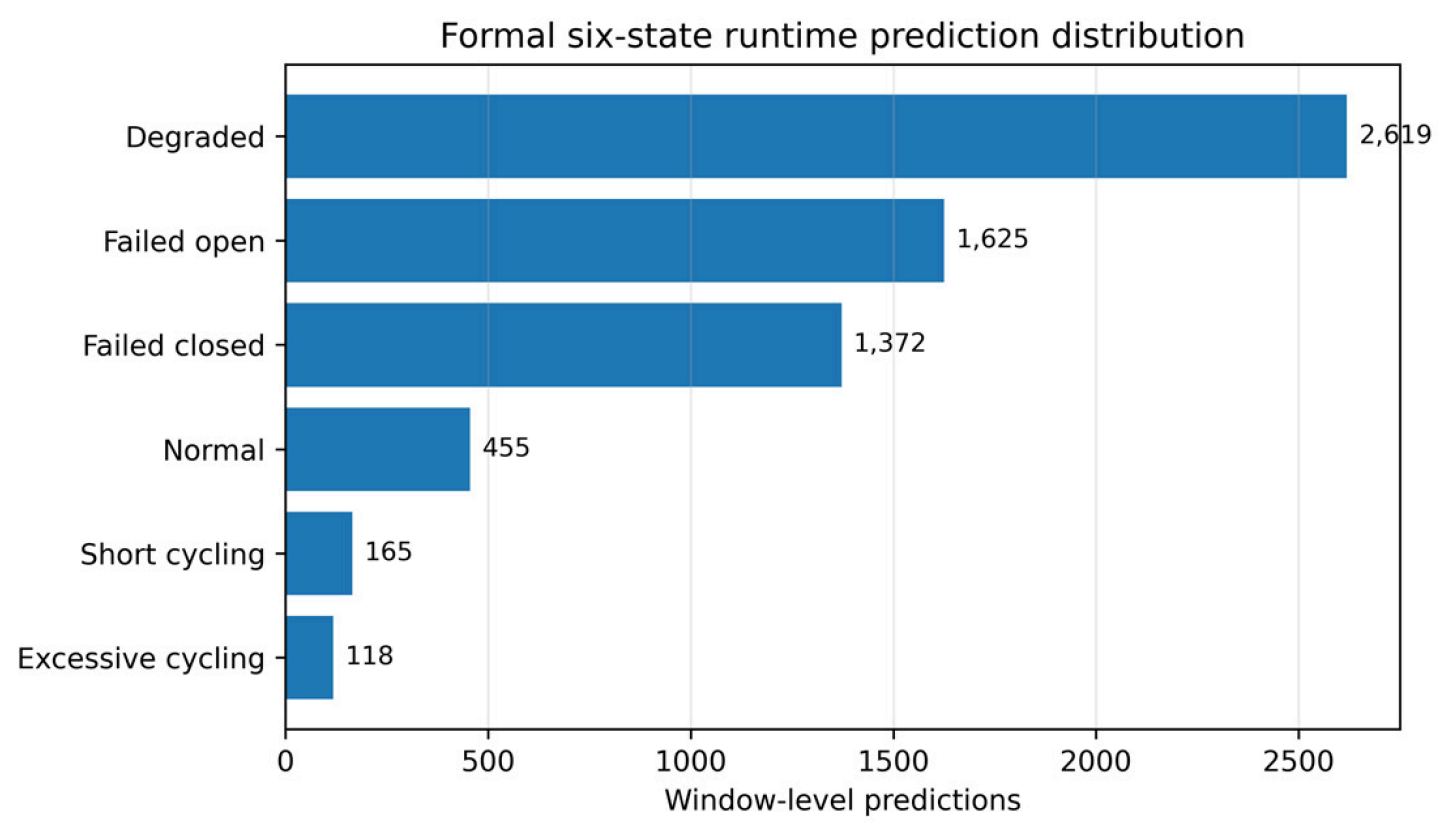
Distribution of formal six-state predictions for the 6354 current-scope runtime records.

**Figure 11:**
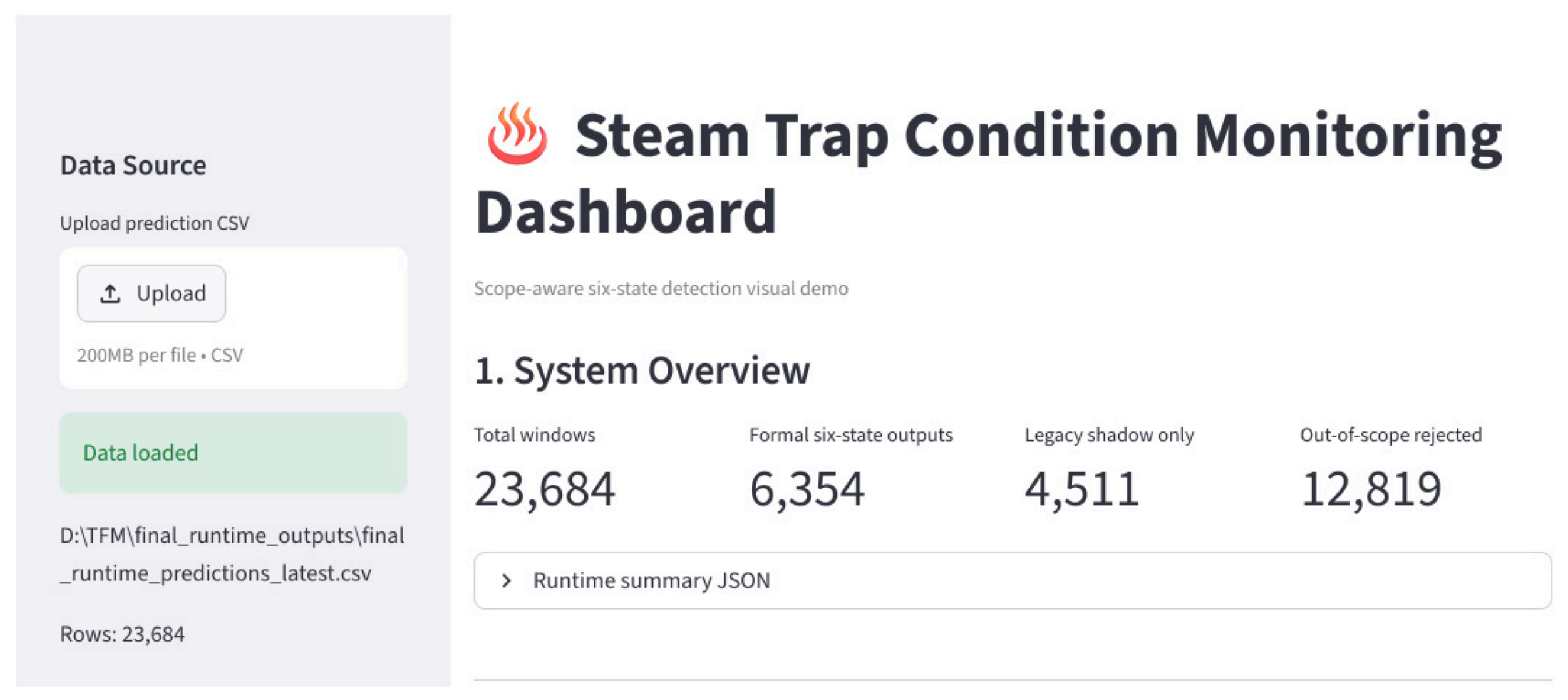
Prototype dashboard overview showing the runtime data source, system summary, and scope-aware record counts. The dashboard processes the runtime output generated by the monitoring pipeline [15].

**Figure 12:**
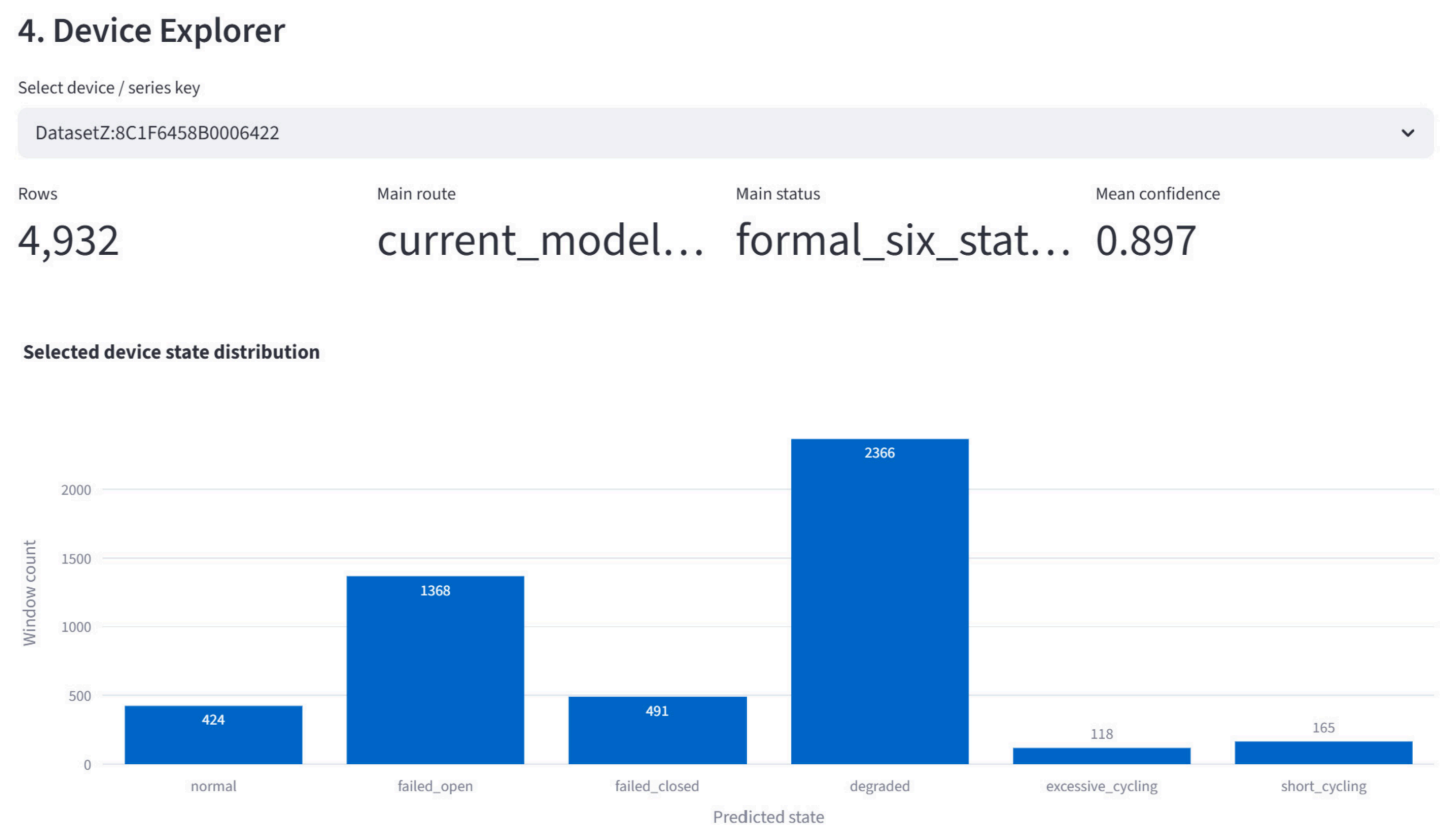
Device Explorer view for a selected device, showing the number of available windows, model/scope status, mean confidence, and the distribution of predicted condition states [15].

**Figure 13:**
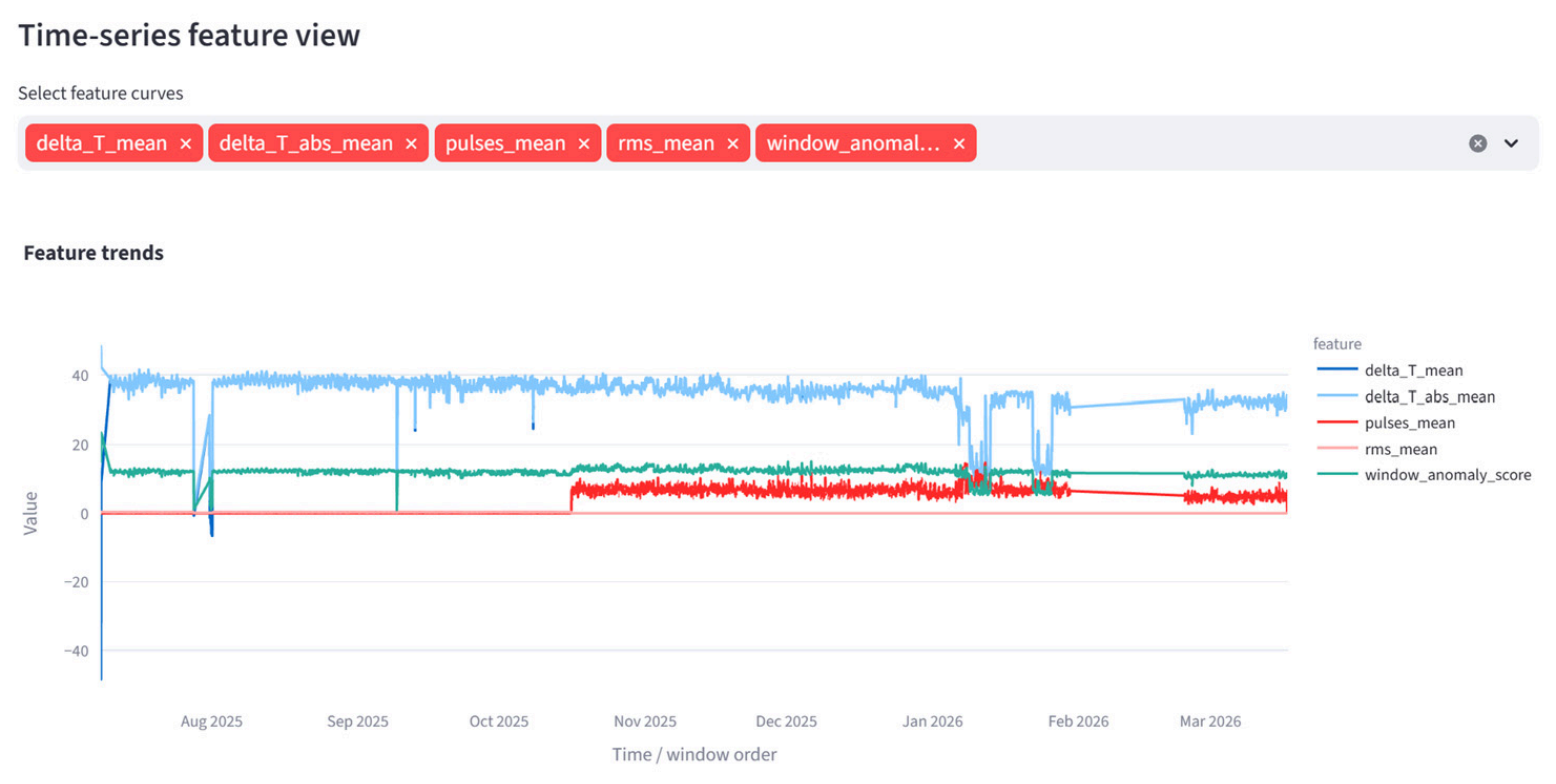
Device-level time-series feature inspection view, showing selected temperature and acoustic/derived features over time for visual interpretation of the model inputs [15].

**Table 1:** Steam-trap families, operating principles, and monitoring implications.

Trap Family	Dominant Physical Mechanism	Monitoring Implications
Mechanical	Density/condensate level	Temperature and continuous or semi-continuous discharge patterns are relevant
Thermodynamic	Flow/pressure dynamics	Pulse, cycle, and ultrasonic behaviour are particularly informative
Thermostatic	Temperature response	Inlet/outlet temperature difference and thermal transients are important

**Table 2:** Engineering condition categories and their qualitative sensor signatures.

State	Physical Interpretation	Typical Sensor Evidence
Normal	Expected operating behaviour	Stable thermal response and trap-specific acoustic/cycling pattern
Failed-open	Continuous or excessive live-steam passage	Hot outlet, persistent ultrasonic activity, loss of normal cycling
Failed-closed	Blocked or insufficient condensate discharge	Hot inlet/cold outlet tendency, low acoustic activity, absence of expected cycles
Degraded	Intermediate abnormal behaviour	Irregular temperature, acoustic or cycling patterns
Excessive cycling	Conceptually, cycle frequency above the expected operating range	Elevated pulse activity; correspondence to complete operating cycles remains unverified
Short cycling	Conceptually, unusually short cycle durations or inter-cycle intervals	Rapid repeated pulse activity; cycle timing and class boundary remain unverified

**Table 3:** Summary of thermoelectric characterisation previously reported in [7].

Condition	Hot-Side T	Ambient T	Reported Result
Maximum open-circuit voltage	170 °C	15 °C	3.687 V
Maximum generated power	170 °C	15 °C	1.034 W
Characterisation range	50–170 °C	15–40 °C	5 ambient conditions
Temperature step	10 °C	—	20 min stabilisation/step

**Table 4:** Window-level feature groups and their engineering interpretation.

Feature Group	Examples	Physical Information
Temperature	temperature, inlet temperature, outlet temperature, ΔT, |ΔT|	Thermal state and thermal balance
Ultrasound/acoustic	RMS, mean RMS, band ratio, peak value, peak frequency	Flow- and leakage-related acoustic activity
Pulse/cycling	pulse count, pulse frequency, mean/std pulses	Dynamic opening/closing behaviour
Statistical windows	mean, standard deviation, minimum, maximum, range, rolling statistics	Short-term operating variability
Metadata/quality	device ID, mode, diagnostics, missing-value flags	Data validity and device context

**Table 5:** Reported validation and test performance of the three candidate classifiers.

Model	Validation Accuracy	Validation Macro-F1	Test Accuracy	Test Macro-F1
Random Forest	0.9717	0.8030	0.9581	0.7267
Extra Trees	0.9496	0.7171	0.9403	0.4810
Histogram Gradient Boosting	0.9958	0.9550	0.9853	0.8822

**Table 6:** Reported performance across training, validation, and test blocks.

Period	Accuracy	Macro-F1	Interpretation
Training	1.0000	1.0000	Fit to training data
Validation	0.9958	0.9550	Model selection period
Final chronological test	0.9853	0.8822	Later unseen period

**Table 7:** Record counts assigned to predefined runtime scope categories.

Runtime Category	Records	Share
Current scope/formal prediction	6354	26.8%
Legacy/shadow inference	4511	19.0%
Out of scope/rejected	12,819	54.1%
Total	23,684	100%

**Table 8:** Distribution of predicted engineering categories for current-scope records.

Predicted State	Windows
Degraded	2619
Failed-open	1625
Failed-closed	1372
Normal	455
Short cycling	165
Excessive cycling	118

**Table 9:** Comparison of related studies and the present workflow by sensing, dataset, and validation scope.

Study	Sensing or System Focus	Dataset and Trap Context	Reference and Validation	Metrics and Comparison with the Present Study
Kim et al. [1]	Sensor and contextual time-series features; Transformer-based diagnosis	Aluminium-processing and food-manufacturing facilities; trap-population equivalence with the present study is not established	Engineer-supported labelling, including disassembly of selected suspect traps	Diagnostic evaluation uses a different reference basis; scores are not directly comparable with pseudo-label agreement
Li et al. [2]	Steam-trap acoustic-emission denoising	Acoustic signal experiments; no matched trap population or dataset	Evaluation of reconstructed signal quality	Signal-to-noise ratio and reconstruction error address denoising, not six-state classification
Jiang et al. [4]	Thermoelectric self-powered wireless sensing	Industrial waste-heat application; no matched steam-trap classification dataset	Thermoelectric and system performance experiments	Energy-related results provide hardware context, not validation of the present classifier
Giuliano et al. [5]	Energy-aware battery-free LoRaWAN communication	Photovoltaic-powered devices and network scenarios, rather than a steam-trap fault dataset	Consumption characterisation and network simulation	Energy and packet-delivery measures address communication design, not diagnostic validity
Present study	Precomputed thermal, acoustic, and pulse/cycling descriptors; tree-based classification	One facility, two current-scope monitoring-device series; physical trap inventory and operating context incompletely documented	Rule-informed pseudo-labels; reported time-block evaluation without verified device-disjointness	Accuracy and macro-F1 measure label agreement; class-level evidence, uncertainty, and independent diagnostic validation remain unavailable

**Table 10:** Comparison of the previous steam-trap platform [7] and the present analytical workflow.

Dimension	Previous Steam-Trap Work	Present Work	Scientific Implication
Power	TEG-powered, batteryless node	Same autonomous energy concept	Enables continuous sensing without primary battery
Sensing	Temperature + ultrasound	Temperature + ultrasound + derived cycling features	Multimodal condition representation
Output	Leak detection/abnormality	Six-state condition categories	Richer maintenance information
Analytics	Rule/feature interpretation	Tree-based ML + leakage reduction	Nonlinear multimodal classification
Evaluation	Experimental device characterisation	Chronological, robustness, and scope-aware evaluation	Closer to deployment-oriented assessment
Validation	Demonstrated sensing; limited confirmed faults	Engineering rule labels; independent validation still needed	Defines the current scientific boundary

## Data Availability

The industrial steam-trap monitoring data used in this study were provided by Alternative Energy Innovations S.L. (AEInnova). The raw datasets (DatasetI and DatasetZ) are not publicly available due to confidentiality restrictions associated with the industrial data. The implementation code, trained-model package, scope-routing configuration, post-processing rules, and dashboard prototype are publicly available in the project GitHub repository at: https://github.com/1822971148-ux/steam-trap-predictive-maintenance (accessed on 10 July 2026). The public repository does not contain the confidential raw industrial datasets.
